# Engineered Methionine Adenosyltransferase Cascades for Metabolic Labeling of Individual DNA Methylomes in Live Cells

**DOI:** 10.1021/jacs.4c06529

**Published:** 2024-06-29

**Authors:** Liepa Gasiulė, Vaidotas Stankevičius, Kotryna Kvederavičiu̅tė, Jonas Mindaugas Rimšelis, Vaidas Klimkevičius, Gražina Petraitytė, Audronė Rukšėnaitė, Viktoras Masevičius, Saulius Klimašauskas

**Affiliations:** † Institute of Biotechnology, Life Sciences Center, Vilnius University, LT-10257 Vilnius, Lithuania; ‡ Institute of Chemistry, Faculty of Chemistry and Geosciences, Vilnius University, LT-03225 Vilnius, Lithuania

## Abstract

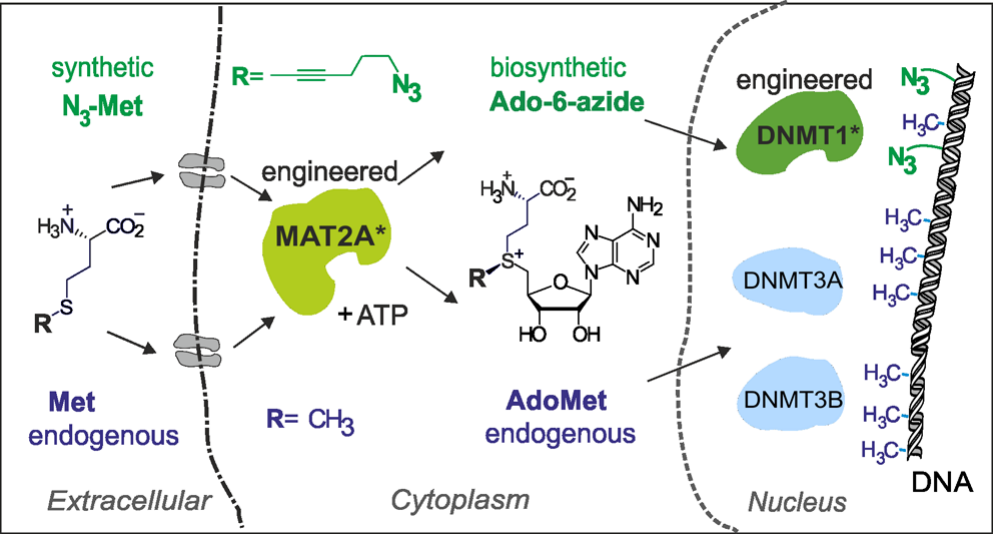

Methylation, a widely occurring natural modification serving diverse regulatory and structural functions, is carried out by a myriad of *S*-adenosyl-l-methionine (AdoMet)-dependent methyltransferases (MTases). The AdoMet cofactor is produced from l-methionine (Met) and ATP by a family of multimeric methionine adenosyltransferases (MAT). To advance mechanistic and functional studies, strategies for repurposing the MAT and MTase reactions to accept extended versions of the transferable group from the corresponding precursors have been exploited. Here, we used structure-guided engineering of mouse MAT2A to enable biocatalytic production of an extended AdoMet analogue, Ado-6-azide, from a synthetic methionine analogue, *S*-(6-azidohex-2-ynyl)-l-homocysteine (N_3_-Met). Three engineered MAT2A variants showed catalytic proficiency with the extended analogues and supported DNA derivatization in cascade reactions with M.*Taq*I and an engineered variant of mouse DNMT1 both in the absence and presence of competing Met. We then installed two of the engineered variants as MAT2A-DNMT1 cascades in mouse embryonic stem cells by using CRISPR-Cas genome editing. The resulting cell lines maintained normal viability and DNA methylation levels and showed Dnmt1-dependent DNA modification with extended azide tags upon exposure to N_3_-Met in the presence of physiological levels of Met. This for the first time demonstrates a genetically stable system for biosynthetic production of an extended AdoMet analogue, which enables mild metabolic labeling of a DNMT-specific methylome in live mammalian cells.

## Introduction

**Figure 1:**
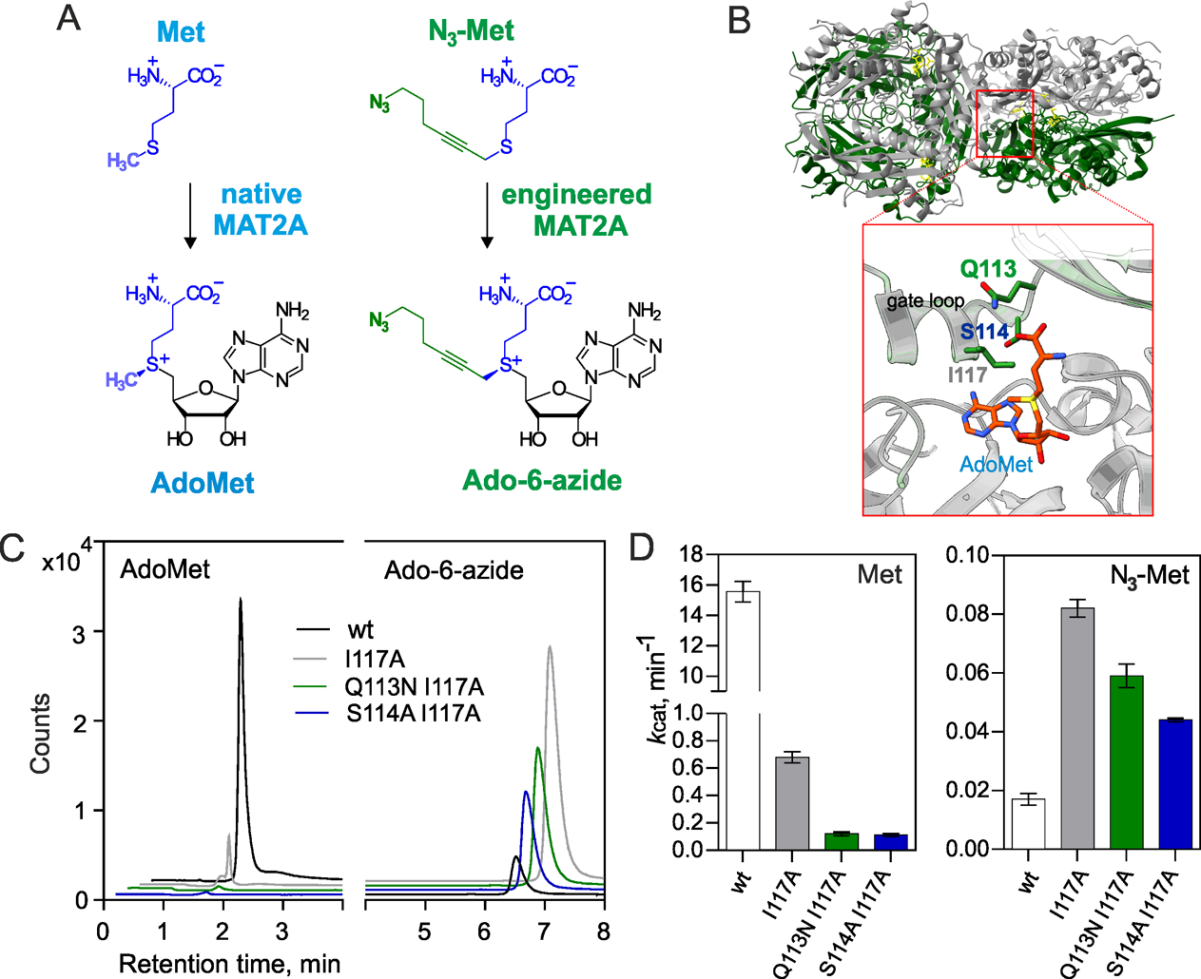
Engineering MAT2A for enhanced catalytic activity with extended methionine analogues. (A) Biological (left) and engineered (right) pathways for enzymatic production of AdoMet and its extended analogue Ado-6-azide, respectively. (B) Ribbon model (upper) of a dimeric human MAT2A reaction complex (PDB entry: 5A1I); inset shows gating loop residues Q113, S114, and I117 around the bound product AdoMet. (C) Representative HPLC–MS/MS chromatograms of biocatalytically produced cofactors AdoMet and Ado-6-azide. (D) Steady-state turnover rates *k*_cat_ of MAT2A variants with Met and N_3_-Met substrates. Error bars denote SEM of 3 measurements.

*S*-Adenosyl-l-methionine (AdoMet) is a ubiquitous methyl donor for a vast variety of biological methylation reactions catalyzed by methyltransferases (MTases).^1,2^ AdoMet is produced from l-methionine (Met) and ATP by a family of multimeric methionine adenosyltransferases (MAT), among which the MAT2A protein is the key catalytic unit in most mammalian cells. Due to the biological and biomedical significance of the transmethylation reactions involving DNA, RNA, and proteins, chemical tools based on MTase-directed functionalization and labeling of their biomolecular targets using synthetic AdoMet analogues with extended allylic and propargylic side chains were developed.^3−6^ However, poor membrane permeability and limited stability of AdoMet and its analogues make their application for cell-based research challenging. Since methionine analogues can be taken up by the cell through amino acid transporters,^7^ similar strategies based on a bump-and-hole engineering^8,9^ or substrate promiscuity were applied for chemo-enzymatic biosynthesis of AdoMet cofactors using matching methionine analogue and MAT pairs.^7,10−17^ Yet, in-cell studies exploiting MAT2A for the synthesis of AdoMet analogues were typically limited to rather short transferable moieties (3–6 carbon units) and conditions of a (nearly) complete depletion of endogenous methionine.^7,18−20^ The latter limitation puts a severe hurdle on studies of epigenetic mechanisms, since methionine deprivation leads to dramatic alterations of DNA, RNA, and histone methylation,^21−24^ protein expression profiles, and even induction of stem cell differentiation.^25,26^ Recently, selective tracking of the DNMT1 activity in genome-edited mouse embryonic stem cells (ESC) has been demonstrated, in which entry of an AdoMet analogue, Ado-6-azide,^27^ was achieved using pulse electroporation.^28^ However, due to potential negative effects of electrical discharge and limited applicability of electroporation on the tissue or organism levels, we sought to develop a genetically stable system for biosynthetic production of Ado-6-azide cofactor in the presence of physiological levels of methionine (Figure 1A) and thus enable mild metabolic DNMT-directed DNA labeling in live cells.

## Results and Discussion

Toward this goal, our first step was to produce a suitable mouse MAT2A mutant that shows enhanced selectivity toward the extended Met analogue, *S*-(6-azidohex-2-yn-1-yl)-l-homocysteine (N_3_-Met). The N_3_-Met analogue was chemically synthesized from l-homocystine and 6-azidohex-2-yn-1-ol using an improved method featuring transient *N*-Boc protection of the α-amino group to preclude potential side reactions during the *S*-alkylation step (Scheme S1). This strategy avoids costly and scale-limiting reversed-phase chromatography purification mandatory for one-pot reduction/alkylation approaches^11,29^ and thus affords a hundred-milligram scale synthesis. Previous engineering of human MAT2 indicated that expanding the substrate binding pocket by an I117A replacement enhances the acceptance of extended methionine analogues.^29^ However, cell-based studies with the human MAT2A I117A variant in a cascade pathway with engineered protein methyltransferases showed selective target labeling only upon complete depletion of endogenous methionine.^7,20^ In an attempt to further reduce the inherent preference of the enzyme for the natural substrate, we turned our attention to the gate loop (residues 113–131)—a flexible polypeptide flanking the active site.^30−32^ Gln113 is the only residue of the gate loop that directly interacts with the bound Met. Ser114 interacts with the adenine ring of bound ATP *via* a water bridge and is thought to stabilize the closed helical conformation of the loop (Figure 1B). Altogether, the wt and three engineered variants I117A, Q113N I117A, and S114A I117A were produced (Figure S1) for *in vitro* studies of the MAT2A substrate selectivity.

After confirming stereoselective enzymatic *S*-adenylation of Met and the extended analogue N_3_-Met (Figure S2A), we characterized the mutants by determining their steady-state kinetic parameters using high-performance liquid chromatography–mass spectrometry/MS (HPLC–MS/MS) quantitation of the accumulated cofactors (Table 1 and Figures 1C,D and S2).

**Table 1:** Apparent *k*_cat_ and *K*_m_ Values^a^ of Mouse MAT2A Variants with Met and N_3_-Met Substrates

		*k*_cat_		*K*_m_				
MAT2A variant	substrate	min^–1^	Mut/wt	mM	Mut/wt	*k*_cat_/*K*_m_, min^–1^ mM^–1^	Met selectivity	relative N_3_-Met selectivity
wt	Met	15.56 ± 0.68	(1)	0.020 ± 0.003	(1)	778	4.7 × 10^4^	(1)
	N_3_-Met	0.017 ± 0.002	(1)	1.03 ± 0.23	(1)	0.0165	(1)	
I117A	Met	0.679 ± 0.041	1/23	0.645 ± 0.023	32	1.05	12	4 × 10^3^
	N_3_-Met	0.082 ± 0.003	4.8	0.92 ± 0.10	0.9	0.089	(1)	
Q113N I117A	Met	0.119 ± 0.013	1/131	1.40 ± 0.56	70	0.085	2.1	2.4 × 10^4^
	N_3_-Met	0.059 ± 0.004	3.5	1.44 ± 0.27	1.4	0.041	(1)	
S114A I117A	Met	0.111 ± 0.011	1/140	1.40 ± 0.41	70	0.079	2.7	1.7 × 10^4^
	N_3_-Met	0.044 ± 0.0006	2.5	1.518 ± 0.043	1.5	0.029	(1)	

a Errors denote ± SEM (*n* = 3).

Our studies found that the *k*_cat_(Met) values of the MAT2A variants were remarkably reduced (23-fold for I117A and 131–140-fold for Q113N I117A and S114A I117A), while the turnover rates for N_3_-Met were slightly enhanced (4.8-fold, 3.5-fold, and 2.5-fold, respectively) as compared with the native type. The I117A mutant thus rendered the fastest conversion with both substrates (Figure S2C). Moreover, the mutants showed a ∼50-fold increase in *K*_m_ values for Met (from 0.02 to 0.6–1.4 mM for wt and the mutants, respectively), which now became comparable with those observed for N_3_-Met (0.9–1.5 mM). This observation suggested that the catalytic binding of the precursors in the active site became fairly independent of the size of the chain. Altogether, the N_3_-Met *vs* Met selectivity of the MAT2A mutants defined as the ratio of *k*_cat_/*K*_m_ improved by approximately 4 orders of magnitude (Table 1).

**Figure 2:**
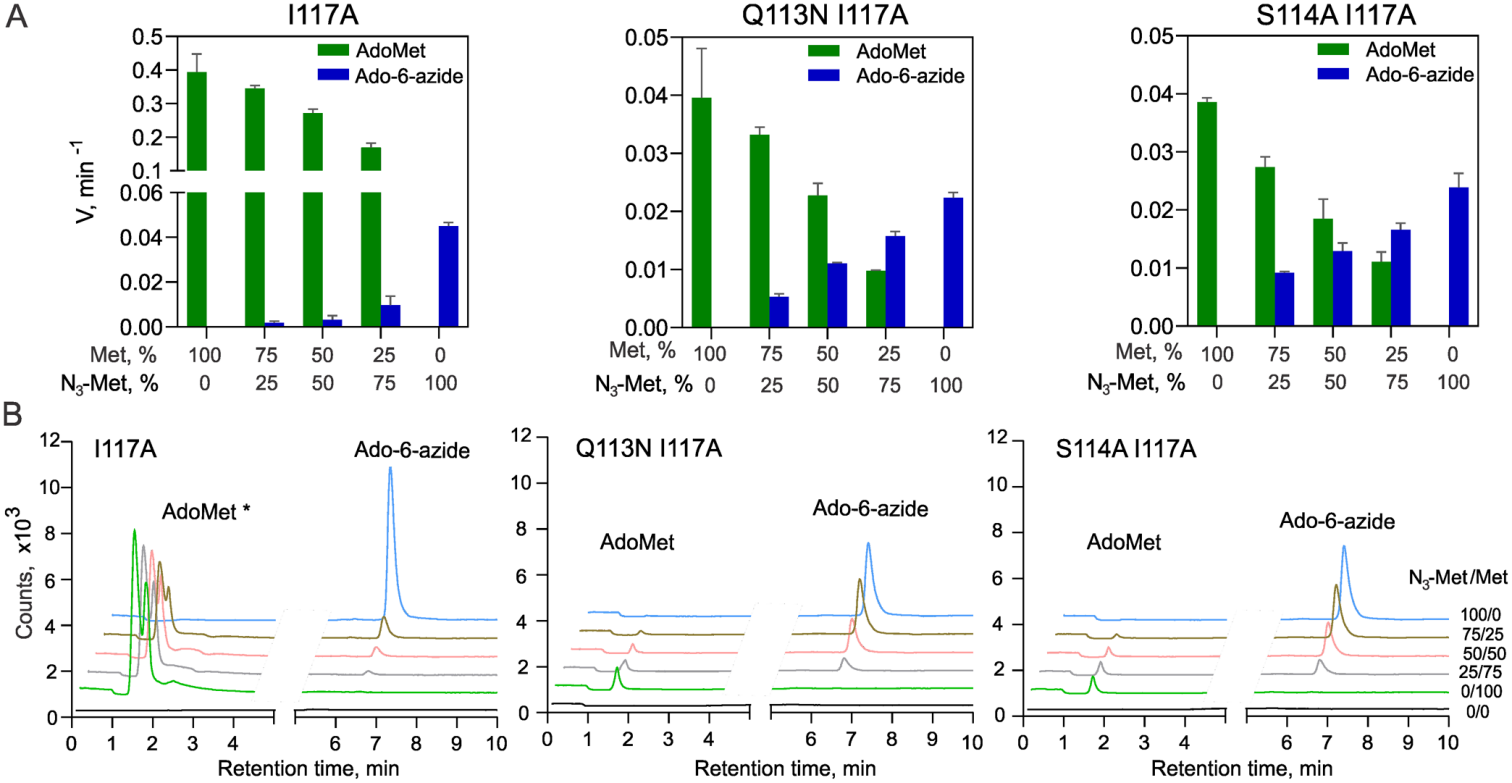
Methionine analogue selectivity of the engineered MAT2A variants. (A) Apparent catalytic rates of AdoMet and Ado-6-azide production by the MAT2A variants in reactions with varied ratios of Met and N_3_-Met (1 mM total concentration); error bars denote the SD of 2–3 determinations. (B) HPLC–MS/MS chromatograms of the biocatalytic reactions depicting cofactor-specific ion transitions for AdoMet and Ado-6-azide. *S,*S*-AdoMet, when produced in high amounts by WT (not shown) or the I117A (left panel) MAT2A variant, often eluted as a distorted or double chromatographic peak (Figure S2B).

We further assessed the substrate selectivity of the MAT2A mutants *in vitro* under conditions of direct competition between N_3_-Met and Met supplied at varied molar ratios. In the presence of equimolar amounts of the two substrates, Ado-6-azide synthesis by the variant I117A was significantly reduced (14-fold) as compared to the original uncontested rate, whereas both double mutants showed a much smaller reduction (∼2-fold) of the transalkylation rate in the presence of AdoMet (Figure 2A,B), and given their 3–4-fold advantage in the production of the desired cofactor under these conditions, they seemed to be superior candidates for further experiments. However, at a higher N_3_-Met/Met ratio (0.75/0.25 mM), the I117A mutant showed only slightly slower Ado-6-azide formation than did the Q113N I117A and S114A I117A variants. Altogether, it turned out that all three variants offered features that seemed valuable under certain conditions, and thus all three were examined in further trials.

**Figure 3:**
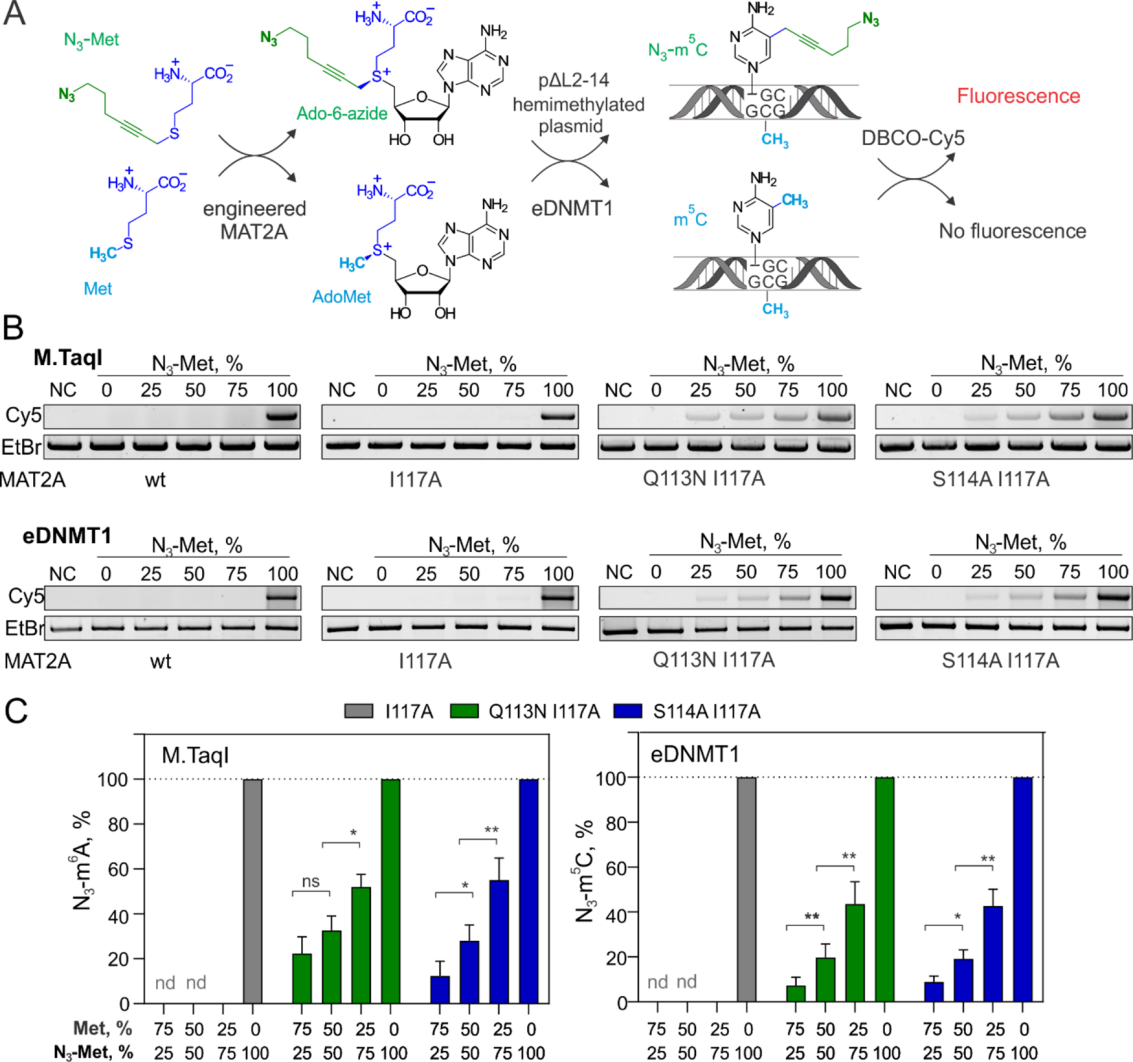
Sequence-specific modification of DNA by MAT2A-DNA methyltransferase cascades with the methionine analogue N_3_-Met in the presence of competing Met. (A) Experimental approach for the analysis of DNA modification in cascade reactions involving engineered MAT2A variants and engineered DNMT1 (eDNMT1). (B) Representative analyses of DNA N_3_-tagging in cascade reactions with M.TaqI (upper) and eDNMT1 (lower) at defined Met and N_3_-Met ratios (shown in mol %). NC, control reaction, without N_3_-Met/Met and protein. (C) Incorporation of N_3_-tags in DNA (determined as the % of Cy5 fluorescence intensity obtained with 100% N_3_-Met) by MAT2A-DNA methyltransferase cascades at distinct N_3_-Met/Met ratios. Error bars denote the SD of 2–3 determinations; nd, not detectable; ns, not significant.

In the next step, we examined the capacity of the MAT2A variants to work in cascade reactions with an adenine-specific MTase, M.TaqI, which proved competent in DNA labeling with a wide spectrum of AdoMet analogues.^10,33^ The results showed that all engineered variants rendered full DNA protection after 1 h of incubation with either substrate, except for the WT enzyme (Figure S3). Further, we examined the MAT2A mutants in one-pot transalkylation cascades with M.TaqI or the engineered variant of mouse DNMT1^28^ (eDNMT1) under conditions of direct precursor competition with methionine (at various N_3_-Met/Met ratios) (Figures 3 and S4). The DNA modification reactions were monitored by click-tagging the transferred azide groups with a DBCO-Cy5 fluorophore. In both cases, only weak signals were observed with the I117A variant, but substantial modification levels were observed in cascade reactions with the doubly substituted variants (Figure 3B,C). Altogether, this identified two MAT2A variants (Q113N I117A and S114A I117A) that can efficiently utilize the synthetic Met analogue in the presence of endogenous Met in enzymatic cascades with DNA MTases *in vitro*.

To install these bioorthogonal labeling cascades in mammalian cells, we made genomic replacements of the corresponding codons (I117A, Q113N/I117A, and S114A/I117A) in exon 4 of the *Mat2a* gene (Figure S5) of our previously derived biallelic *Dnmt1*^N1580A^ mouse embryonic stem cell line.^28^ Monoallelic knock-in (KI) substitutions were incorporated by using a Cas9 nickase-RT-based search-and-replace editing system.^34^ Restriction analysis of the corresponding PCR amplicons identified that the editing efficiency was about 15% in the case of both I117A and S114A/I117A (Figure S5) with the other *Mat2a* allele retained as the active native type. Unexpectedly, despite several attempts, we failed to identify mESC clones containing the desired Q113N codon replacement and thus continued our studies with the derived cell lines encoding the I117A and S114A/I117A variants. Three randomly picked clones from each KI cell line were subjected to sequencing of the *Mat2a* locus, and selected individual clones of each type were analyzed for allele-specific gene expression and cellular protein levels (Figure S6B,C).

**Figure 4:**
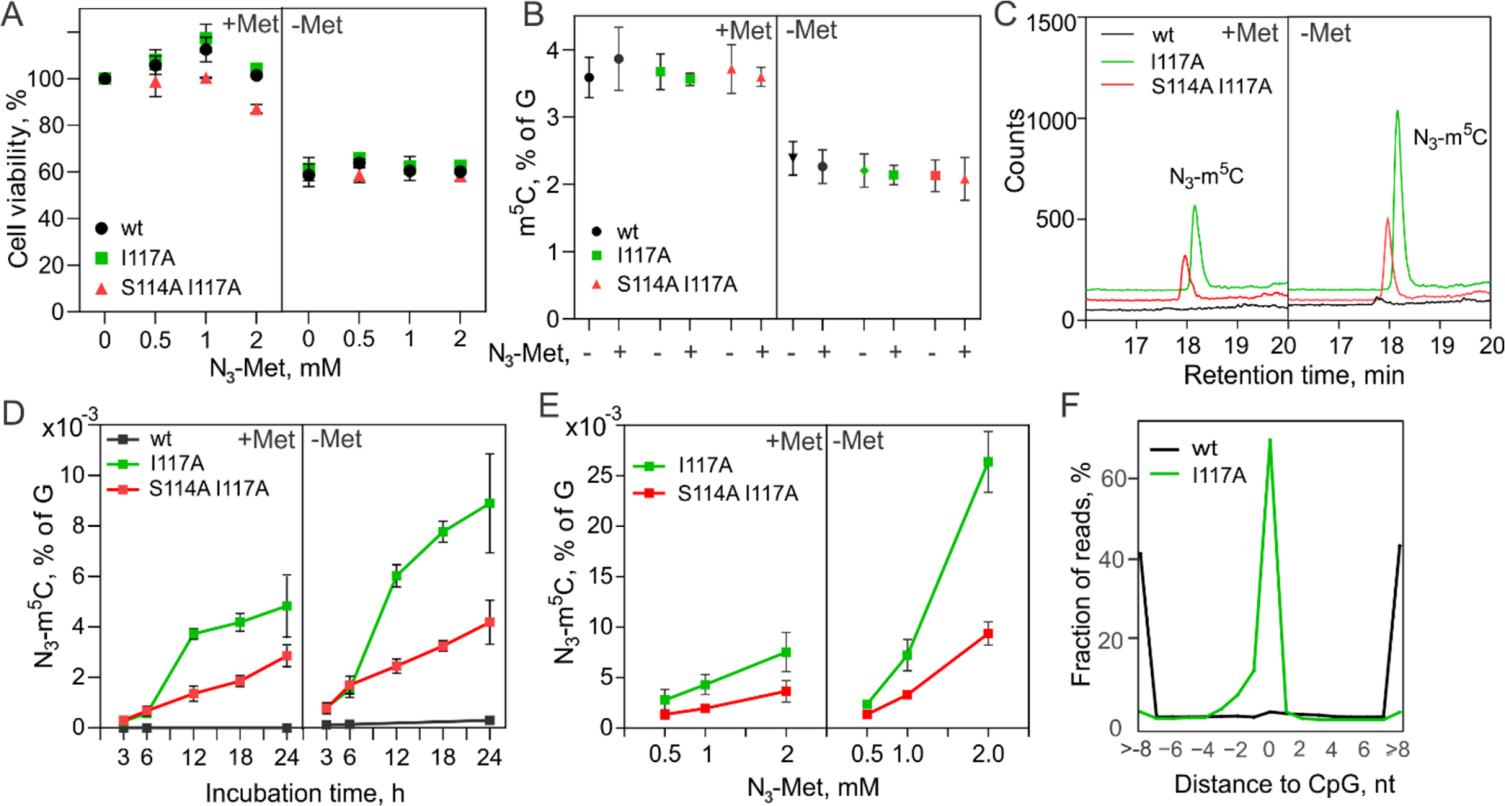
Metabolic tagging of genomic DNA by the engineered MAT2A-DNMT1 cascade in live ES cells. (A) Cell viability after 24 h incubation with N_3_-Met, as determined by the MTT assay. (B) Global methylation (m^5^C levels normalized to dG) in mESCs after 24 h incubation with/without 1 mM N_3_-Met in LIF medium containing 0.2 mM (+Met) or none (−Met) methionine. (C) HPLC–MS/MS chromatograms of N_3_-m^5^C in genomic DNA from engineered ESCs after 24 h incubation with 1 mM N_3_-Met. (D) Time-course of metabolic DNA cytosine modification (N_3_-m^5^C levels normalized to dG) in mESCs after incubation with 1 mM N_3_-Met. (E) Dose dependence of metabolic DNA modification levels in mESCs after 24 h incubation with N_3_-Met. (F) Proximity of the metabolic tagging sites (determined as read start positions in TOP-seq libraries) to genomic CpG sites; nt, nucleotide. Error bars denote the SD of 3 determinations.

Finally, we examined the behavior of these embryonic cell lines upon exposure to N_3_-Met in the presence of physiological levels (0.2 mM) or in the absence of added Met in LIF medium (Figure 4). In the absence of Met, we observed a 40% decrease in the viable cell numbers after a 24 h exposure, as determined by the MTT assay (Figure 4A); since no dead cells were identified, Met deprivation likely inhibited cell division. Further addition of N_3_-Met to the cell medium at 0.5 to 2 mM concentration had no apparent impact on cell viability. In the presence of Met, a slight reduction in the active cell count was observed only upon 24 h of incubation of S114A/I117A cells with 2 mM N_3_-Met. We, therefore, conclude that N_3_-Met shows little, if any, toxicity to the cells under our experimental conditions. We then looked at the global genomic levels of m^5^C and N_3_-m^5^C using HPLC–MS/MS analysis. Although Met deprivation resulted in a significant reduction of genomic m^5^C, as expected,^25,35−37^ the engineered cell lines showed nearly identical m^5^C levels to the native-type cells, indicating that the monoallelic *Mat2a* substitutions had virtually no effect on DNA methylation (Figure 4B). Remarkably, we found a clearly detectable concentration-dependent and time-dependent accumulation of genomic N_3_-m^5^C in both engineered cell lines upon administration of N_3_-Met; moreover, less than a 2-fold reduction of the genomic azide tagging occurred in the presence of competing Met as compared to Met-free conditions (Figure 4C–E). Control cell lines with either one or both wt genes showed none or a barely detectable N_3_-m^5^C signal in all cases, indicating that both engineered genes were required for the tagging reactions *in vivo* (Figures S7 and 4C,D). A high degree of bioorthogonality of the system is also attested by the lack of detectable formation of azide-tagged nucleotides in total RNA or labeled proteins in I117A cells as compared to control WT cells (Figures S8 and S9).

In contrast to a clearly superior activity of the S114A/117A variant in direct competition assays *in vitro* (Figure 3), the I117A cells unexpectedly showed twice as high DNA labeling efficiency as compared to the S114A/I117A cells! One possible reason for the inverse activity order of the MAT2A variants could be the fact that all three generated mutants showed millimolar *K*_m_(Met) values (Table 1), suggesting that the active site of the enzyme would not be saturated at a 0.2 mM concentration of Met in cell medium. This implies that the enzyme would still be capable of binding and turning over added N_3_-Met along with the natural substrate. Given its 2-fold higher transalkylation activity in the absence of Met *in vitro* (Figures 2A and S2), the observed 2-fold higher DNA labeling activity by the I117A variant appears reasonable. Another important factor to consider is the multimeric structure of the enzyme and the presence of the wt *Mat2A* allele along with the mutant in the heterozygotic KI cells. This apparently leads to the assembly of tetrameric forms containing distinct combinations of the two MAT2A variants *in vivo*, as opposed to the monogenic form of the enzyme produced *in vitro* from bacterial cells. As multiple catalytic centers are assembled at subunit interfaces, the effects of possible intermonomer interactions on the ability to accept methionine analogues are unclear at this point. Moreover, this could further be modulated by interactions with the regulatory MAT2B subunit, which was shown to alter the *K*_m_ for methionine or even restore the activity of the S114A mutant *in vitro*.^31^ Overall, the benefit of the additional S114A mutation in this context remains elusive.

The density of intracellular metabolic tagging in I117A cells (24 h of incubation with 2 mM N_3_-Met) was about 400 times (0.008 *vs* 3.5%) or 90 times (0.025 *vs* 2.2%) below the endogenous levels of m^5^C under plus or minus Met conditions, respectively (Figure 4E). The tagging density can likely be increased further by feeding with higher concentrations of the cofactor analogue. To verify the specificity of the DNA labeling, the *in vivo* generated “chemical footprint” was subjected to genomic mapping using the TOP-seq technique,^28^ which generates sequencing reads originating at azide-tagged nucleotides. TOP-seq libraries (∼20 million mapped reads per biological replicate) were prepared from the engineered N_3_-Met-treated ESCs (Figure S10). Consistent with the known sequence-specificity of DNMT1, we found that >90% of the read start positions were at or within 2 nucleotides of a CpG site (Figure 4F), thereby identifying 1.5 million unique modified sites in the genome with an average 12–16-fold read coverage (Figure S10).

In retrospect, it appears somewhat puzzling why a similarly engineered hMAT2A (I117A variant) in cascade with an engineered protein lysine MTase (G9A or GLP1) afforded detectable labeling of target sites only upon depletion of endogenous Met from the cell medium.^7^ While a direct comparison is hardly possible at this point, some of the most obvious differences between the two systems are as follows: (a) different MAT2A enzymes (human *vs* mouse), although quite similar, may differ in certain aspects; (b) different MAT2A expression systems (viral vector *vs* chromosomal) lead to different expression profiles of the enzyme and possibly downstream changes in cellular homeostasis; (c) different host cell lines used (HEK293T cells and mESC) are very different in many aspects (organism, cell type); (d) different targeting MTases (protein lysine MTases *vs* mouse DNMT1) may have different abundances, activity profiles, or availabilities of unmodified target sites during the experimental window; (e) different Met analogues with different/transferable groups (allylic *vs* propargylic) may have distinct cell permeability, *in cellulo* stability or acceptance by enzymatic cascades, *etc.* Overall, these observations reflect the complexity of the AdoMet-producing machinery and its regulatory networks, illustrating the level of uncertainty associated with the behavior of *in vitro*-characterized systems in live mammalian cells.

## Conclusions

This work for the first time demonstrates a functional, chromosomally expressed MAT2A-DNMT cascade in mammalian cells that permits selective chemical tracking of individual DNMT-specific methylomes under native conditions (presence of endogenous nutrients and normal DNA methylation levels). As opposed to labeling techniques that depend on Met deprivation or electroporation-driven cell permeabilization, this noninvasive treatment with a stable nontoxic Met analogue offers new possibilities for *in vivo* studies of epigenetic signaling on the cellular, tissue, and whole organism levels. Given the high structural similarity of AdoMet-dependent MTases, this approach can in principle be extended to study other DNA, RNA, or protein methylation pathways in a variety of eukaryotic systems.

## Supporting Information Available

Purification of engineered MAT2A protein (Figure S1); HPLC-MS/MS analysis of enzymatic *S*-adenylation of Met or N_3_-Met catalyzed by native and engineered I117A, Q113N I117A, and S114A I117A variants of MAT2A (Figure S2); time-course analysis of DNA modification by MAT2A-M.TaqI enzymatic cascades (Figure S3); DNA modification by MAT2A-M.TaqI cascades with the methionine analogue N_3_-Met in the presence of competing Met *in vitro* (Figure S4); derivation of the Mat2a I117A, Q113N I117A, S114A I117A, and Dnmt1 N1580A knock-in (KI) murine embryonic stem cell line (Figure S5); Dnmt1 and Mat2a gene expression and protein levels in engineered embryonic stem E14TG2a cell lines (Figure S6); HPLC-MS/MS analysis of genomic cytosine modification (N_3_-m^5^C) in control mESC lines encoding wt DNMT1 and engineered variants of MAT2A (Figure S7); HPLC-MS/MS analysis of total RNA (Figure S8); non-specific protein labeling in live cells treated with methionine analogue N_3_-Met (Figure S9); bioinformatic analysis of Dnmt-TOP-seq sequencing libraries (Figure S10); raw ^1^H and ^13^C NMR and IR spectra for all synthetic compounds (PDF)
