# Supplementary material for: Engineered Methionine Adenosyltransferase Cascades for Metabolic Labeling of Individual DNA Methylomes in Live Cells
Source: J Am Chem Soc. 2024 Jun 29;146(27):18722–9. doi: 10.1021/jacs.4c06529 (PMC11240257; doi:10.1021/jacs.4c06529)
Supplement: Supplementary file 1 — ja4c06529_si_001.pdf [file ja4c06529_si_001.pdf]

SUPPORTING INFORMATION

Engineered methionine adenosyltransferase cascades for metabolic labeling of individual DNA methylomes in live cells

Liepa Gasiulė,[1] Vaidotas Stankevičius,[1] Kotryna Kvederavičiūtė,[1] Jonas Mindaugas Rimšelis,[1] Vaidas Klimkevičius,[1,2] Gražina Petraitytė,[1,2] Audronė Rukšėnaitė,[1] Viktoras Masevičius[1,2] and Saulius Klimašauskas[1,*]

[1]Institute of Biotechnology, Life Sciences Center, Vilnius University, LT-10257 Vilnius, Lithuania

[2]Institute of Chemistry, Faculty of Chemistry and geosciences, Vilnius University, LT-03225 Vilnius, Lithuania

*Correspondence: saulius.klimasauskas@bti.vu.lt

Table of Contents

SUPPORTING FIGURES 3

METHODS 13

CHEMICAL SYNTHESIS of S-(6-azidohex-2-yn-1-yl)-L-homocysteine (Scheme S1) 21

SUPPORTING REFERENCES 25

^{1}H and ^{13}C NMR and IR spectra of the synthesized compounds 26

SUPPORTING FIGURES

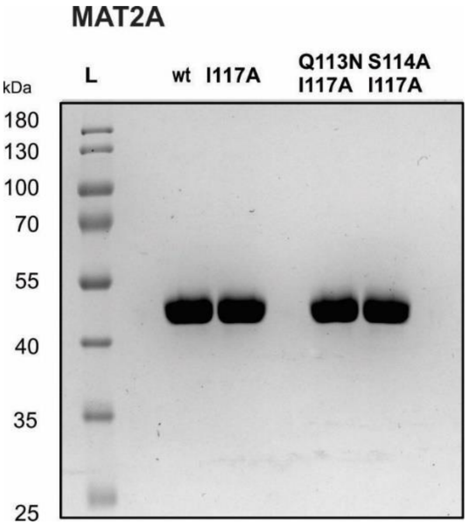

Figure S1. Purification of engineered MAT2A protein. Recombinant N-terminal His_6-tagged MAT2A variants were expressed in *E. coli* BL21 (DE3) and purified using immobilized Ni^{2+} affinity chromatography. 2 µg of each purified protein were subjected to analysis on 12% SDS-PAGE along with a prestained protein ladder, stained with Coomassie Brilliant Blue and scanned. Theoretical mass of the wt protein is 45184 Da.

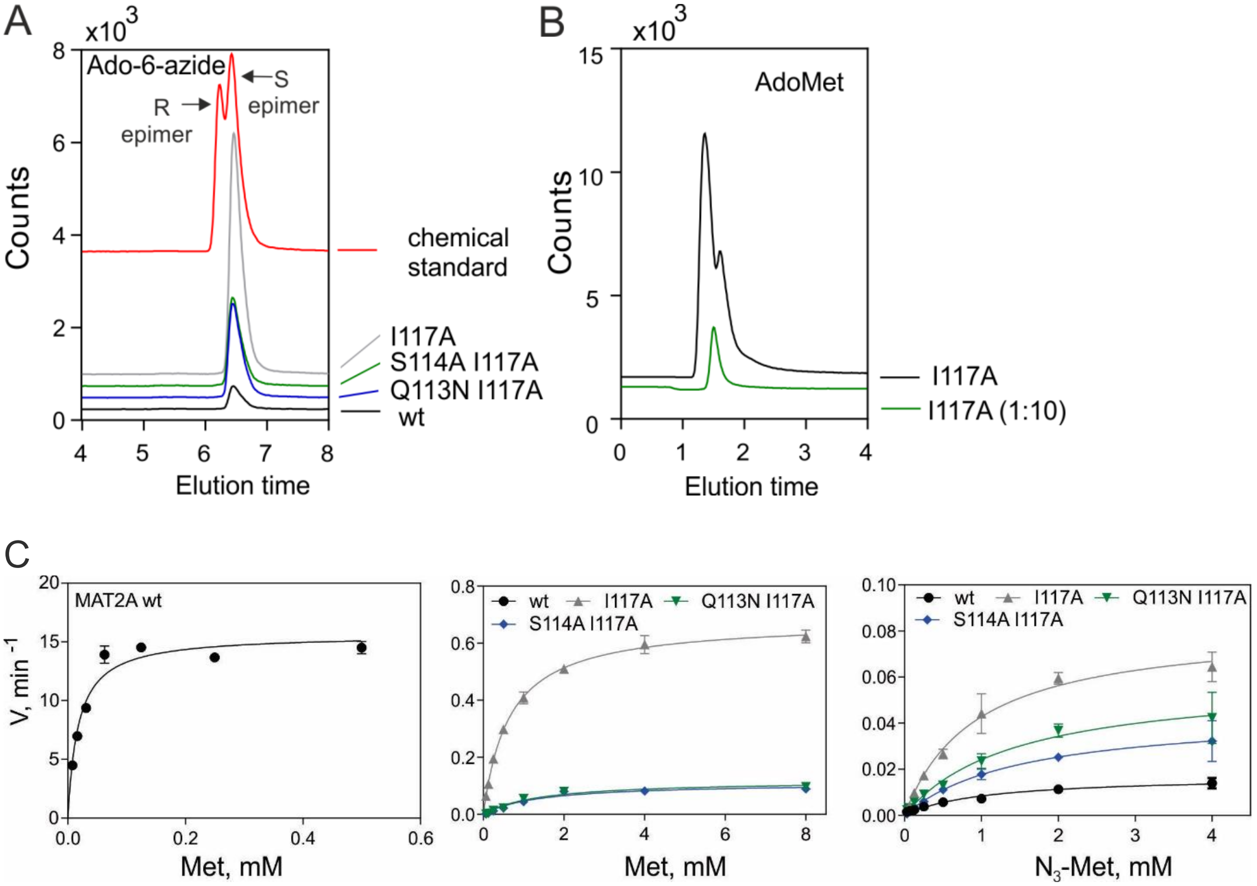

Figure S2. HPLC-MS/MS analysis of enzymatic *S*-adenylation of Met or N_3-Met catalyzed by native and engineered I117A, Q113N I117A, and S114A I117A variants of MAT2A. A) Chromatographic comparison of Ado-6-azide produced by stereospecific *S*-adenylation of N_3-Met by specified MAT2A variants with synthetic Ado-6-azide obtained by chemical *S*-azidoalkylation of *S*-adenosyl-L-homocysteine comprising a mixture of *S,S*- and *R,S*- isomers.[1] B) Chromatographic appearance of enzymatically produced S,S-AdoMet in MAT2A I117A-catalyzed reactions. Shown are traces from direct injection of a reaction aliquot (upper trace) and a 1:10 diluted sample (lower trace). C) Michaelis–Menten kinetics of MAT2A variants with Met or N_3-Met. Reactions were carried out at specified Met or N_3-Met concentrations, 1 mM ATP and MAT2A (0.25–10 µM wt, 5–10 µM I117A, 10 µM Q113N I117A, 10 µM S114A I117A) in reaction buffer (25 mM Tris, at pH 7.4, 5 mM $MgCl_2$, and 50 mM KCl). Samples were incubated for 5–30 min at 37 °C, quenched with HCOOH, and reaction products AdoMet and Ado-6-azide were quantified using HPLC/ESI-MS/MS as described. Determined reaction velocities (V) were fitted to a standard Michaelis–Menten equation. Error bars denote SD of 3 determinations. Derived kinetic parameters are presented in Table 1.

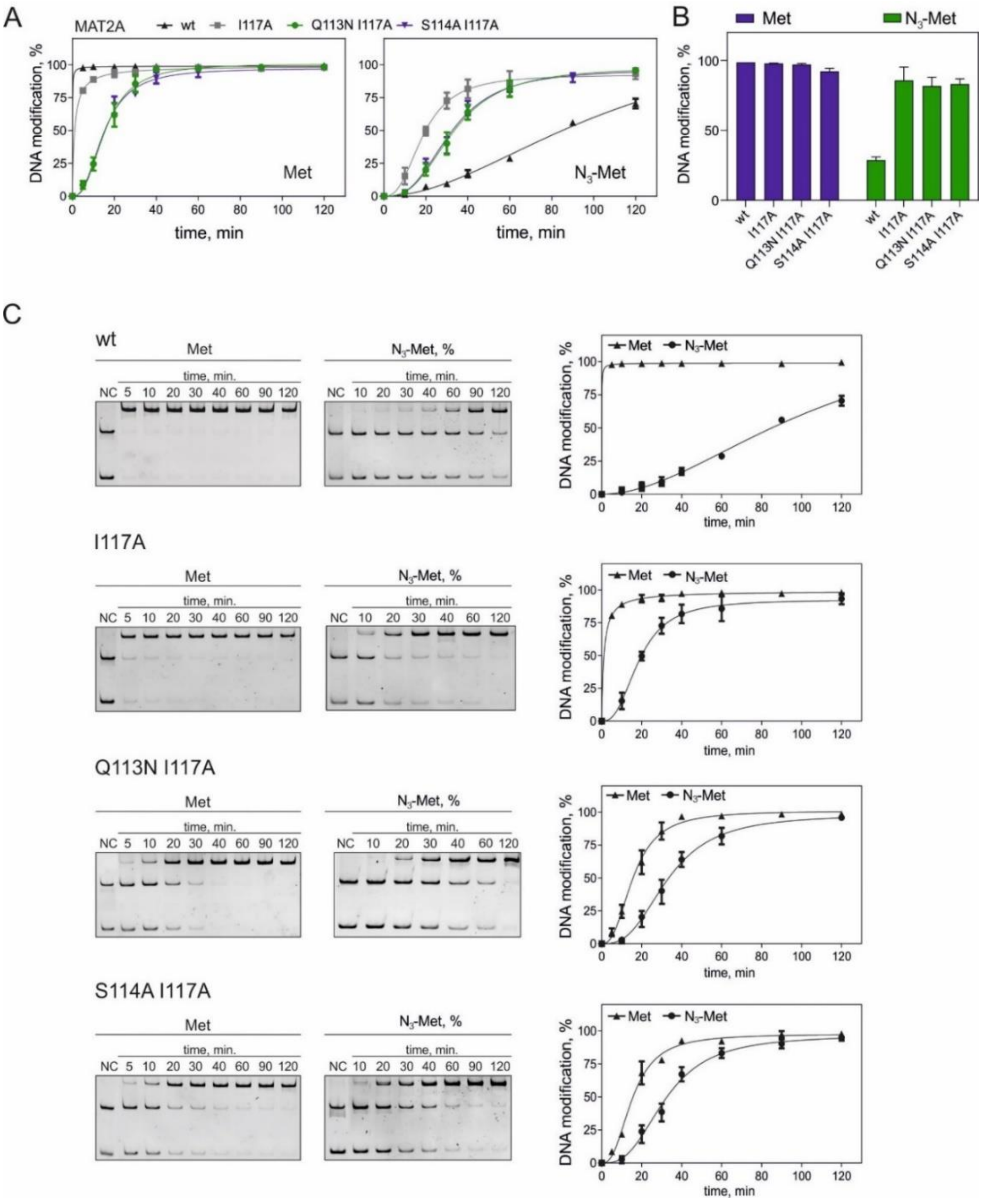

Figure S3. Time-course analysis of DNA modification by MAT2A-M.TaqI enzymatic cascades. Reactions containing 20 µM MAT2A, 0.8 µM M.TaqI, 0.05 µM DNA (376 bp fragment with a single TCGA site), 1 mM ATP and 0.25 mM Met or N_3-Met were incubated for specified periods at 37 °C and then heat-inactivated at 80 °C. Extent of DNA modification was determined by incubation with restriction endonuclease R.TaqI, which cleaves unmodified TCGA sites, followed by analysis of DNA fragmentation products by non-denaturing 6% PAGE. A) Kinetics of DNA modification by the enzymatic cascades in the presence of Met (left) and N_3-Met (right). B) Levels of DNA modification after 1 h incubation with Met or N_3-Met. C) Representative analyses of DNA modified by MAT2A-M.TaqI cascades with specified MAT2A variants. NC indicates a control reaction without Met/ N_3-Met. Error bars show SD of 2–3 replicates.

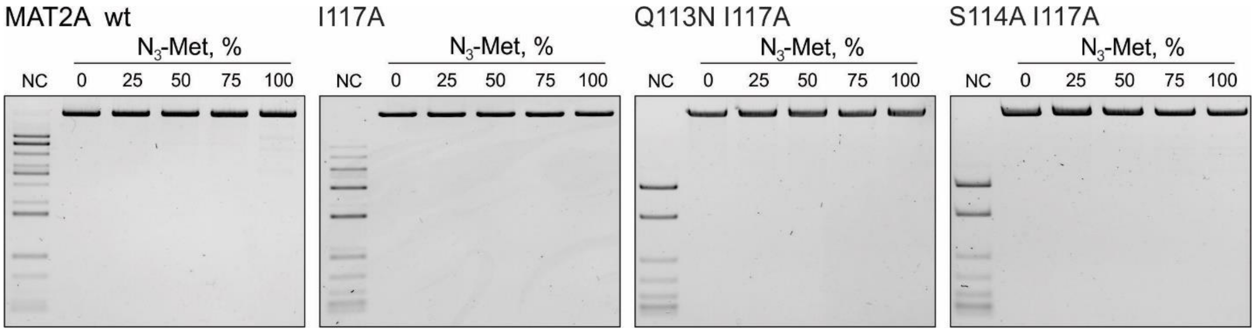

Figure S4. DNA modification by MAT2A-M.TaqI cascades with methionine analog N_3-Met in the presence of competing Met *in vitro.* Reactions containing 20 µM MAT2A, 0.8 µM M. TaqI, 0.5 µg DNA fragment (0.125 µM TCGA sites), 1 mM ATP and 1 mM mixture of N_3-Met and Met as specified were incubated for 18 h at 37 °C. Extent of DNA modification (methylation + azide tagging) at TCGA sites was determined by incubation of samples with R.TaqI and R.NdeI (for plasmid linearization), followed by analysis of DNA fragmentation products on a 1% agarose gel. NC- control reaction with no Met or N_3-Met.

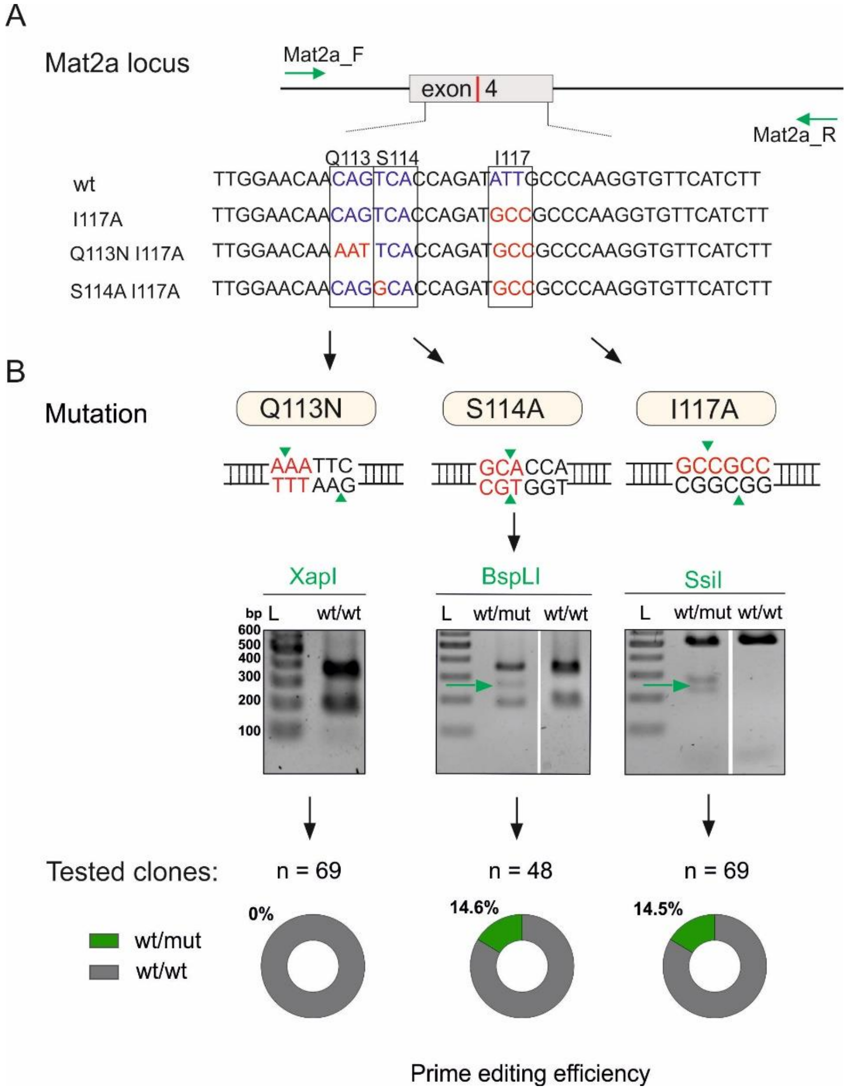

Figure S5. Derivation of the Mat2a I117A, Q113N I117A, S114A I117A and Dnmt1 N1580A knock-in (KI) murine embryonic stem E14TG2a cell line. A) Genomic locus of *Mat2a* exon 4 (encoding residues 110-125). Exon shown as a grey box, lines represent introns; and codon substitutions shown in red; B) Analysis of genomic KI substitutions by cleavage at built-in diagnostic restriction endonuclease sites in the murine embryonic stem Dnmt1 N1580A E14TG2a cell line. Diagnostic 537 bp PCR fragment (amplified from primers shown by green arrows) yields the following fragmentation patterns with WT and engineered alleles:

	WT	Engineered	Mutation
XapI	365 + 169 bp	260 + 105 + 169 bp	Q113N
BspLI	369 + 186 bp	**264** + 186 + 86 bp	S114A
SsiI	504 + 33 bp	**274** + **229** + 33 bp	I117A

DNA fragments indicative of the engineered codons are shown boldface in the table and by green arrows on gels.

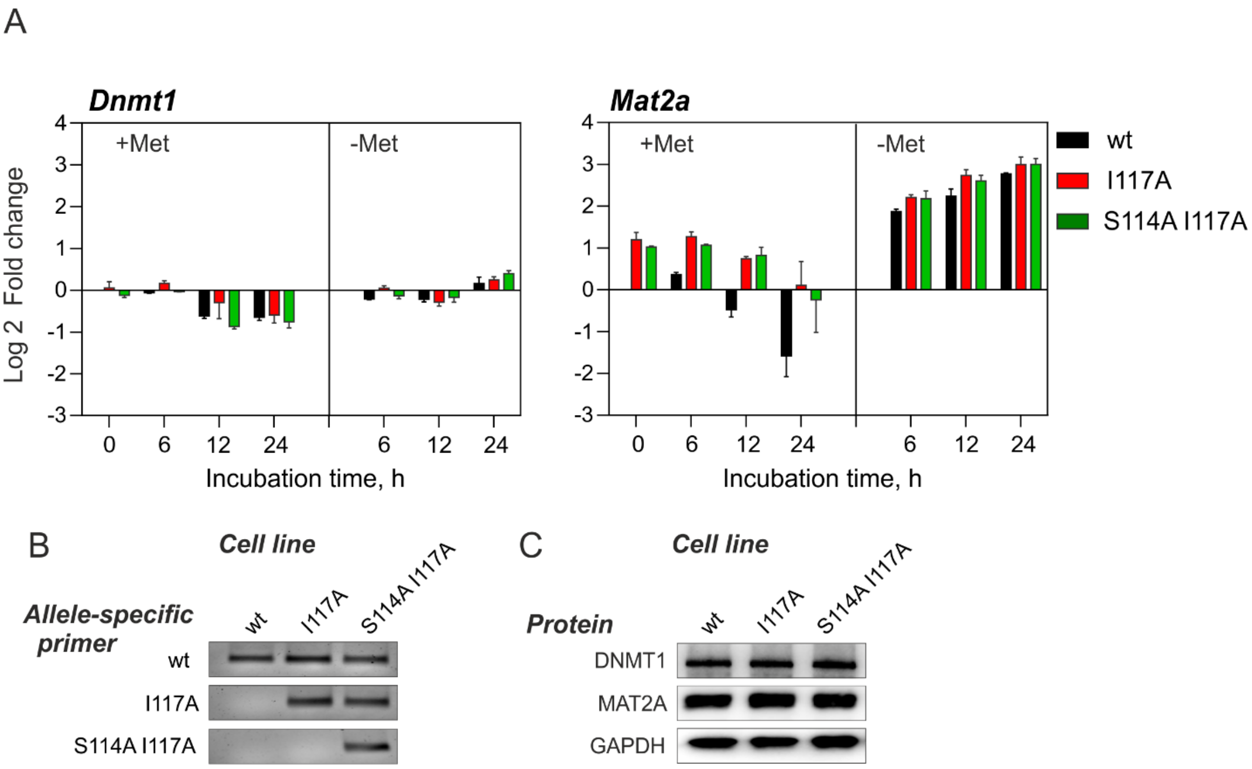

Figure S6. ***Dnmt1*** **and** ***Mat2a*** **gene expression and protein levels in engineered embryonic stem E14TG2a cell lines.** A) *Dnmt1* and *Mat2a* gene expression (mRNA levels) fold changes after 0 h, 6 h, 12 h, 24 h incubation with the presence of N_3-Met with Met or in Met starvation compared to *wt* cell line untreated controls; 2 biological experiments were conducted, GAPDH was used for endogenous control. Error bars denote ± SD. B) Allele-specific mRNA expression in specified cell lines; qPCR products were amplified 30 cycles and separated by electrophoresis in 2% agarose gels. C) Western blot analysis of DNMT1 and MAT2A protein levels in specified cell lines. 3 biological experiments were carried out. No protein changes were identified between cell lines.

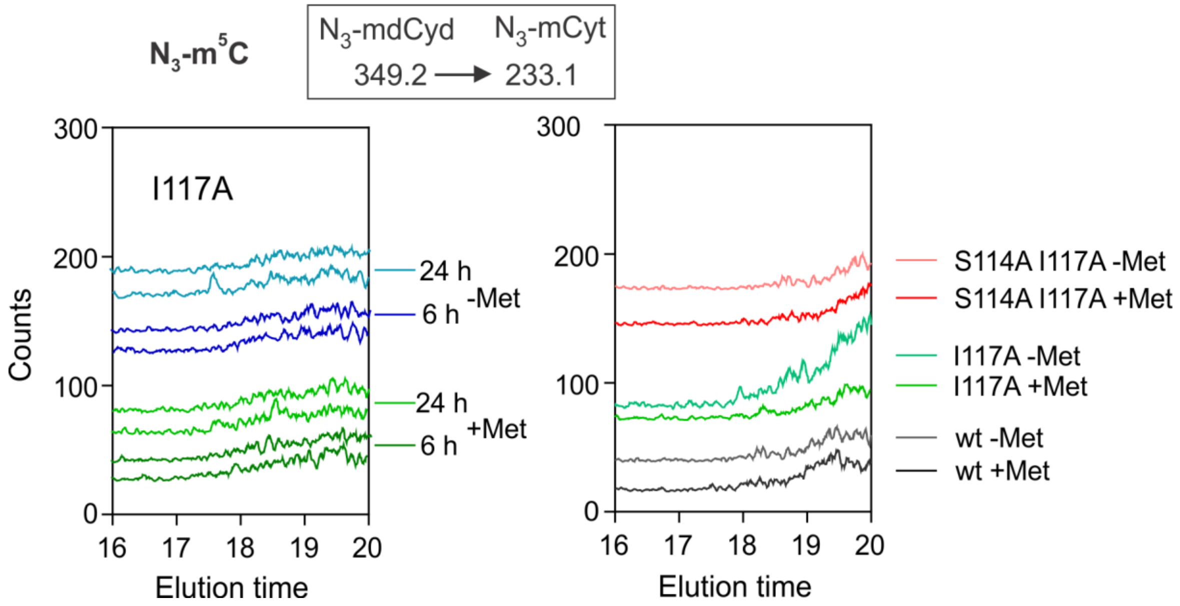

Figure S7. HPLC-MS/MS analysis of genomic cytosine modification (N_3-m^{5}C) in control mESC lines encoding wt DNMT1 and engineered variants of MAT2A. Analysis of DNA isolated from cells encoding the MAT2A I117A variant and DNMT1 wt after 6 h or 24 h treatment with N_3-Met in the presence or absence of Met (shown 2 biological replicates for each condition, left panel) or from cells encoding specified MAT2A variants and DNMT1 N1580A after 24 h incubation with Met or in Met starvation (right panel). Experimental conditions are identical with those used in Figure 4C (see Methods below for details).

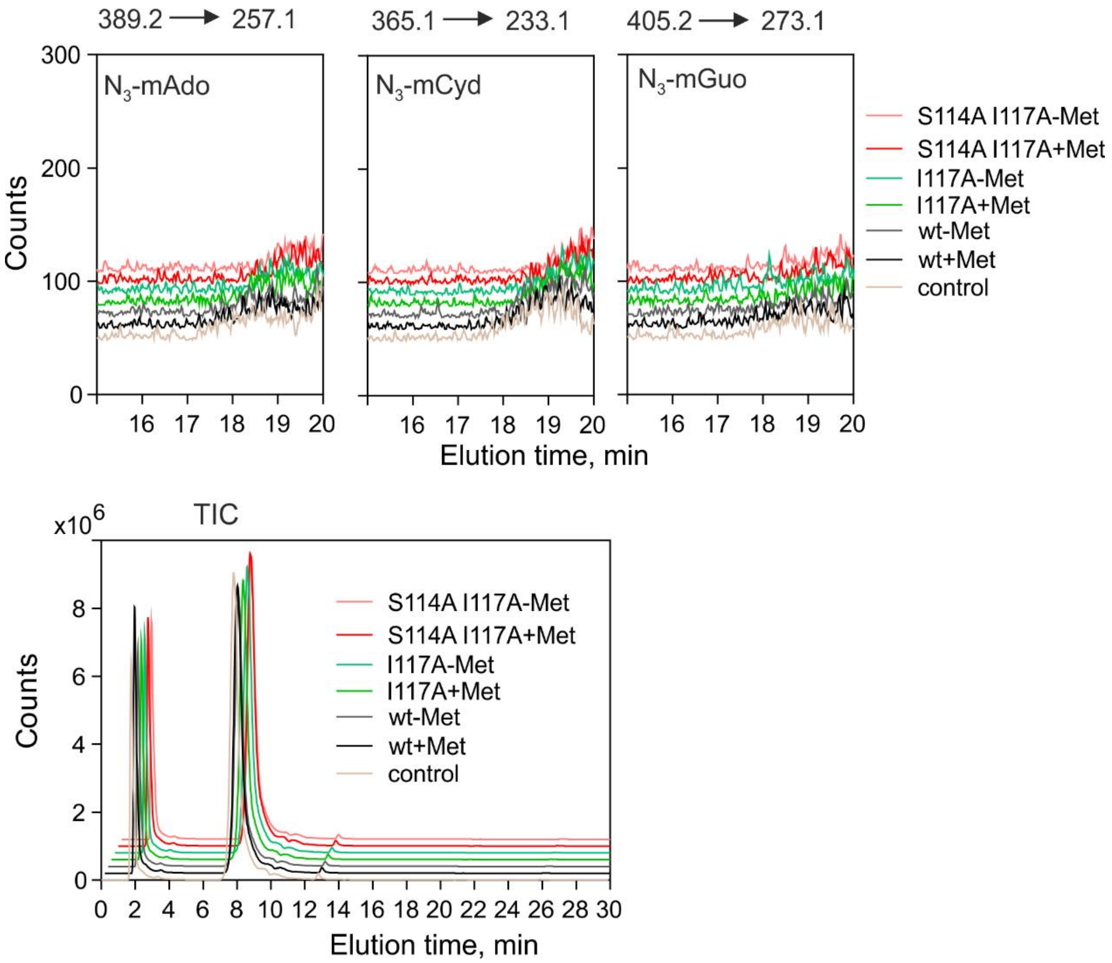

Figure S8. **HPLC-MS/MS analysis of total RNA.** RNA was isolated from E14TG2a cells and treated with 1 mM N_3-Met for 24 h with plus or minus Met in the medium. 1 µg of RNA isolated from each experiment was enzymatically hydrolyzed to nucleosides and injected onto a HPLC-MS/MS system running in MRM mode in which the following ribonucleoside ion transitions were recorded: C 244.1$\rightarrow$112.1, G 282.1$\rightarrow$152.1, mAm 296.1$\rightarrow$150.1, mA 282.0$\rightarrow$150.1, mG 298.1$\rightarrow$166.1, mC 258.1$\rightarrow$126.1, N_3-mA 389.2$\rightarrow$257.1, N_3-mAm 403.2$\rightarrow$257.1, N_3-mC 365.1$\rightarrow$233.1, N_3-mG 405.2$\rightarrow$273.1. Top, chromatograms of individual nucleoside $\rightarrow$ nucleobase ion transitions for theoretical azide-hexynyl-tagged ribonucleosides as shown; control, untreated wt cells. Bottom, TIC chromatograms (counts of all listed ion transitions together) of the same samples. One of 2 biological replicates is shown.

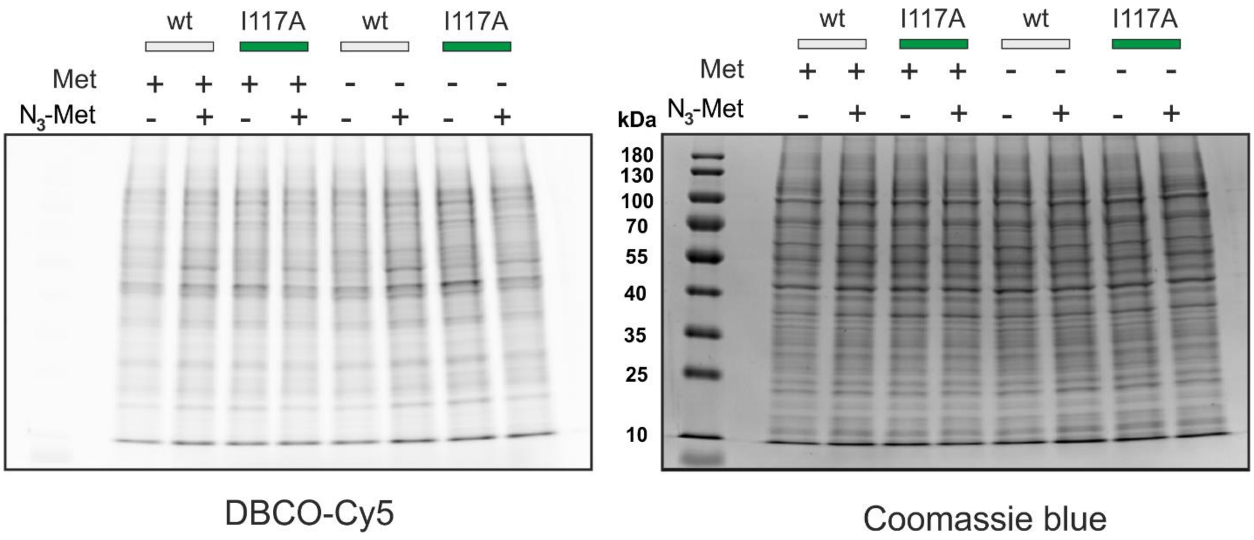

Figure S9. **Non-specific protein labeling in live cells treated with methionine analog N_3-Met.** Wt mouse ESC cells (wt) and engineered Mat2a $^{wt}/^{I117A}$ Dnmt1 $^{N1580A}/^{N1580A}$ cells (I117A) were incubated for 24 h with 1 mM N_3-Met in the presence (+Met) or absence (-Met) of 0.2 mM Met. Cells were lysed with ice-cold RIPA buffer containing EDTA-free protease inhibitor cocktail. Proteins were precipitated with 1:2:3 $CHCl_3/H_2O$/MeOH and subjected to click-labeling with 100 µM DBCO-Cy5 at 37 °C for 1.5 h. Excess of Cy5 dye was removed by repeated protein precipitation. Labeled proteins were resolved by 12% SDS-PAGE and imaged with a fluorescence scanner (left panel); gel was then stained with Coomassie Brilliant Blue and imaged with a density scanner (right panel).

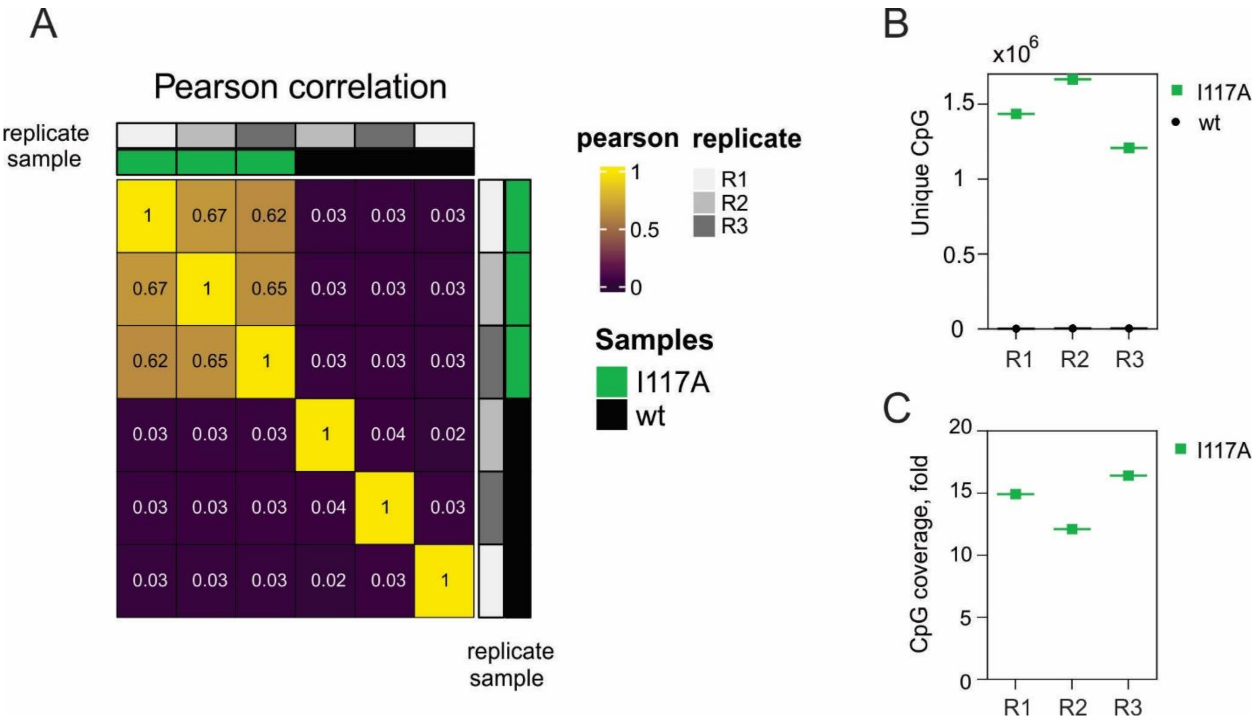

Figure S10. Bioinformatic analysis of Dnmt-TOP-seq sequencing libraries. A) Pearson correlation between the Dnmt-TOP-seq datasets' biological replicates. Correlation heatmap between library replicates generated from normal wt cells (wt) and bearing double cascade mutations Mat2a wt/I117A Dnmt1 N1580A/ N1580A (I117A) mESC. Reads were counted per non-overlapping 2 kb windows resembling an average CGI size. B) Number of captured unique CpG targets in wt and Mat2a wt/I117A Dnmt1 N1580A/ N1580A libraries; C) Average read coverage for identified CpGs, R- replicate.

METHODS

Site-directed mutagenesis of MAT2A

pLTE1 vector encoding N-terminal His_6-tagged MAT2A I117A variant was purchased from Novo Pro. Q113N, S114A replacements, and reversion to native-type I117 were introduced by PCR. Each reaction contained 0.5 µM reverse and forward primer (Metabion, Table S1), 7 ng DNA template, 0.2 mM dNTPs, 1 U Phusion High-Fidelity DNA Polymerase (ThermoFisher Scientific), 5 x HF buffer. The PCR protocol included an initial step of 98 °C for 30 sec, followed by 40 cycles of 98 °C for 10 sec, 70 °C for 30 sec, 72 °C for 90 sec, and then a final step of 72 °C for 5 min. DNA fragments were purified from agarose gel using *GeneJET Gel Extraction Kit* (ThermoFisher Scientific) according to manufacturer recommendations and phosphorylated with PNK (ThermoFisher Scientific). Reactions contained 300 ng DNA, 10 x PNK buffer, and 1 mM ATP and were incubated at 37 °C 20 min, followed by 75 °C, for 10 min. Next, reaction mixtures were then supplemented with 0.5 µl T4 DNA ligase buffer, 0.5 µl 10 mM ATP, 2.5 µl PEG 4000, and 1 µl T4 DNA ligase (ThermoFisher Scientific). Samples were incubated for 1 h at room temperature and ligated DNA fragments were transformed into *E. coli* DH5α cells. Plasmids were extracted using *Gene Jet Plasmid Miniprep* (ThermoFisher Scientific), and the sequences were verified by Sanger sequencing.

Table S1. Primers used for site-directed mutagenesis.

**Primer**	**Sequence, 5'..3'**
wt FW	TCACCAGATGTGGCCCAAGGTGTTCATC
wt REV	CTGTTGTTCCAAGGCAACCAACACAT
Q113N FW	TTGGAACAAAACTCACCAGATGCCGC
Q113N REV	GGCAACCAACACATTACAAGTCTTGTAG
S114A FW	TTGGAACAACAGGCGCCAGATGCCG
S114A REV	GGCAACCAACACATTACAAGTCTTGTAG

MAT2A protein expression and purification

Plasmids containing the MAT2A variants were used for the transformation of *E. coli* BL21 (DE3) cells. Cells were grown in LB medium containing 100 µg/mL ampicillin at 37 °C until the OD_{600} reached 0.6, then cooled to 16 °C, and protein production was induced by adding IPTG to a final concentration of 0.5 mM. After growth for 18 hours at 16 °C 200 rpm, cells were harvested by centrifugation (4 000 x g, 20 min, 4 °C), washed with sterile water (4 000 x g, 20 min, 4 °C) and pellets were stored at -80 °C for further use. Next, cell pellets were resuspended in lysis buffer (20 mM $Na{\cdot}HPO_4$, 500 mM NaCl), 20 mM imidazole, 1 mM PMSF, *cOmplete*™ EDTA-free Protease Inhibitor Cocktail (one tablet to 50 ml buffer), pH=7.4). The cells were lysed with sonication (7 min, 80 mV amplitude, 1 s pulse), the lysate was cleared by centrifugation at 49 000 × g, 4 °C and the supernatant was filtered through a 0.44 µm filter. Protein purification was performed on an ÄKTA purifier. A HisTrap HP column (GE HealthCare) was equilibrated in buffer A (20 mM $Na{\cdot}HPO_4$, 500 mM NaCl, 20 mM imidazole, pH=7.4) the sample was injected and proteins were eluted by an increasing gradient of imidazole, using elution buffer (20 mM $Na{\cdot}HPO_4$, 500 mM NaCl, 250 mM imidazole, pH=7.4). Fractions containing the desired protein were pooled and two dialysis were performed against dialysis buffer (400 mM NaCl, 80 mM KCl, 10 mM $Na{\cdot}HPO_4$, 0.04% 2-

mercaptoethanol, followed by dialysis against storage buffer (80 mM KCl, 10 mM Na·HPO_4, 0.04% 2-mercaptoethanol, 50% glycerol). The dialyzed protein was aliquoted and stored at −20 °C. Protein concentrations were measured by a Bradford assay kit (ROTI®Nanoquant reagent (Roth)) with BSA as standards. 2 µg of proteins were subjected to 12% SDS-PAGE. PageRuler™ Prestained Protein Ladder, 26616, (ThermoFisher Scientific) was used. Gel was stained with Coomassie Brilliant Blue dye (0.09% Brillant Blue R 250, 45% ethanol (96%), 9% acetic acid).

DNMT1 N1580A expression and purification

DNMT1 N1580A was purified as previously described [2].

Determination of kinetic parameters of MAT2A variants

To determine k_{cat} and K_m values of MAT2A proteins the reactions were conducted in a volume 20 µl with saturating 1 mM ATP, at varied concentrations of Met (Sigma) or N_3-Met, wt 0.25–10 µM, I117A 5–10 µM, Q113N I117A 10 µM, S114A I117A 10 µM, in a buffer 25 mM Tris, at pH 7.4, 5 mM $MgCl_2$, and 50 mM KCl. Reactions were incubated 5–30 min at 37 °C and subsequently quenched with 1 µl 1% HCOOH followed by centrifugation (20 000 x *g*, 20 min, 4 °C) to remove the precipitated protein. Samples were diluted and were subsequently integrated with HPLC/ESI-MS/MS system (Agilent 1290 Infinity/ 6410 Triple Quad LC/MS) equipped with a Supelco Discovery HS C18 column (7.5 cm × 2.1 mm, 3 µm) by elution with a linear gradient of solvents A (0.0075 % formic acid in water) and B (0.0075 % formic acid in acetonitrile) at a flow of 0.3 mL/min at 30 °C as follows: 0–5 min, 0 % B; 5–15 min, 10 % B; 15–20 min, 100 % B. Mass spectrometer was operating in the positive ion MRM mode and intensities of cofactor-specific ion transitions were recorded: AdoMet 399.1→ 250.1 and 399.1→ 136.1; Ado-6-azide 506.2→ 250.1 and 506.2→ 136.1. Data were normalized to AdoMet or Ado-6-azide cofactors calibration curves (linear range from 0.8–100 pmol). Assays were repeated in triplicate. Data were analyzed using Agilent MassHunter software and Microsoft Excel. The steady-state kinetic parameters were obtained by fitting the initial velocities (the production of AdoMet or Ado-6-azide vs. the reaction time) against the concentrations of Met or N_3-Met with GraphPad Prism 5 software according to the standard Michaelis–Menten equation. Methionine analog selectivity of the engineered MAT2A variants was determined by incubating reactions at various 0/100, 25/75, 50/50, 75/25, 100/0 N_3-Met/Met molar ratios at a total 1 mM concentration, in the presence of 1 mM ATP, 5 µM I117A, Q113N I117A, S114A I117A in 25 mM Tris, at pH 7.4, 5 mM $MgCl_2$, and 50 mM KCl. Reactions were incubated for 20 min at 37 °C and subsequently stopped with 1 µl 1% HCOOH followed by centrifugation (20 000 x *g*, 20 min, 4 °C). AdoMet and Ado-6-azide cofactors were analysed using HPLC-MS/MS as described above.

Time-course of DNA modification with MAT2A-M.TaqI cascades

For time-course experiments, a 376 bp DNR fragment containing one M. TaqI TCGA target site was amplified from pBR322 plasmid (ThermoFisher Scientific). Reaction contained 0.5 µM reverse and forward primer (pBR322 TaqI FW/REV, Metabion, Table S2), 7 ng pBR322, 0.2 mM dNTPs, 1 U *DreamTaq* DNA Polymerase (ThermoFisher Scientific), 10 × *DreamTaq* buffer. The PCR protocol included an initial step of 98 °C for 30 sec, followed by 35 cycles of 98 °C for 10 sec, 60 °C for 30 sec, 72 °C for 45 sec, and then a final step of 72 °C for 5 min. PGR product was purified using *GeneJet PCR Purification Kit*. One-pot cascade reactions were carried out with 20 µM MAT2A variant, 0.8 µM M. TaqI (NEB), 125 ng DNA fragment (0.05 µM TCGA sites), 0.25 mM Met/N_3-Met, 25 mM Tris-HCl (pH=7.4), 5 mM $MgCl_2$, 50 mM KCl, 1 mM ATP, in a total volume 20 µl. Reactions were incubated for 5, 10, 20, 30, 40, 60, 90, 120 min at 37 °C and inactivated at 80 °C 10 min. Next, samples were treated by 1.5 U Proteinase K (ThermoFisher Scientific) with 0.5% SDS at 55 °C for 1 h, followed by 65 °C for 20 min. DNA fragments were column-purified with *Oligo Clean &*

Concentrator (*Zymo Research),* fragmented with FastDigest TaqI restriction endonuclease (ThermoFisher Scientific) into 132 bp and 244 bp fragments, and analyzed in non-denaturing 6% PAGE gel. The percentage of modified (protected from cleavage) DNA was calculated from 2–3 independent biological replicates.

Table S2. Primers used for PCR fragment amplification.

Primer	Sequence, 5'..3'
pBR322 TaqI FW	ACTGGTCCCGCCACCAAACG
pBR322 TaqI REV	GGCTCCATGCACCGCGAC

DNA modification reactions with MAT2A-M.TaqI cascades at various N_3-Met/ Met ratios

Reactions were carried out with 20 µM MAT2A, 0.8 µM M.TaqI (NEB), 500 ng pBR322 plasmid (containing 7 TaqI targets, DNA targets concentration 0.125 µM), 25 mM Tris-HCl (pH=7.4), 5 mM $MgCl_2$, 50 mM KCl, 1 mM ATP, at various 0/100, 25/75, 50/50, 75/25, 100/0 N_3-Met/Met molar ratios using total 1 mM amino acids concentration, in volume 20 µl. Reactions were incubated overnight at 37 °C, inactivated at 80 °C for 10 min, next samples were treated by 1.5 U Proteinase K with 0.5% SDS at 55 °C for 1 h, followed by 65 °C for 20 min. DNA fragments were purified using *Oligo Clean & Concentrator* (Zymo Research) and samples were divided in two equal fractions. The first fraction was incubated with 5 µM DBCO-Cy5 (Sigma), 25 mM Tris-HCl, pH = 7.4, in a total volume 20 µl at 37 °C for 2 h. Unreacted Cy5 dye was removed using *Oligo Clean & Concentrator* (Zymo Research). Plasmid DNA was linearized by incubation with FastDigest Eco91I ir HindIII (ThermoFisher Scientific) in volume 10 µl and 5 µl of 50% glycerol was added to each sample. Linearized DNA was fractionated on a 1% agarose gel, gel was scanned for Cy5 fluorescence (λ_{ex}=649 nm, λ_{em}= 666 nm) with *ChemiDoc^{TM}* imaging system, followed by ethidium bromide staining. The intensity of Cy5 fluorescence was evaluated with *Image Lab*, and plotted as % of Cy5 fluorescence intensity obtained in reaction with 100% N_3-Met. 2–3 independent biological repeats were carried out.

Preparation of hemimethylated pΔL2-14 plasmid

pΔL2-14 plasmid [3] was transformed into *E.coli* ER2267 cells. One colony was inoculated in 200 ml LB medium and incubated with shaking at 30 °C for 18 h, and then at 37 °C until OD_{600} reached ~0.6. Then IPTG was added to a final concentration of 0.4 mM, cells were cultivated for 2 h at 37 °C and were harvested by centrifugation (4 000 x g, 20 min, 4 °C), washed by water (5 000 x g, 20 min, 4 °C) and pellets were stored at -80 °C for further use. Plasmid DNA was purified using *GeneJET Plasmid Maxiprep Kit* (ThermoFisher Scientific). The methylation level of the substrate pΔL2-14 plasmid was verified by digestion with restriction endonuclease HhaI (ThermoFisher Scientific).

Enzymatic coupled cascade with MAT2A and DNMT1 N1580A with methionine analog N_3-Met and competing Met *in vitro*

The enzymatic cascades with MAT2A and DNMT1 N1580A reactions were performed in volume 20 µl containing – 20 µM MAT2A, 2.6 µM DNMT1 N1580A, 500 ng hemimethylated pΔL2-14 plasmid (containing 92 hemimethylated CGC/Gm^{5}CG sites in the sequence, DNA targets concentration 0.8 µM), 25 mM Tris-HCl (pH=7.4), 5 mM $MgCl_2$, 50 mM KCl, 1 mM ATP, at various 0/100, 25/75, 50/50, 75/25, 100/0 N_3-Met/Met molar ratios using total 1 mM amino acids concentration, in volume 20 µl. Reactions were

incubated for 4 h at 37 °C, and inactivated at 80 °C 10 min, next samples were supplemented by 1.5 U Proteinase K with 0.5 % SDS at 55 °C 1 h, followed by 65 °C 20 min. DNA fragments were purified using *Oligo Clean & Concentrator* (Zymo Research). Plasmids were labeled with 20 µM DBCO-Cy5 (Sigma), 25 mM Tris-HCl, pH = 7.4, in a total volume 20 µl by incubation at 37 °C for 2 h. Unreacted Cy5 dye was removed using *Oligo Clean & Concentrator* (Zymo Research). Next, plasmids were restricted with FastDigest EcoRI and KpnI (ThermoFisher Scientific) in volume 10 µl, 5 µl 50% glycerol was added to each sample and plasmids were fractionated in a 1% agarose gel. The gel was scanned and analyzed as described above.

Embryonic E14TG2a cell line cultivation

Mouse embryonic stem E14TG2a cell line was obtained from the American Type Culture Collection of Authenticated Cell Cultures (ATCC CRL-1821). Mat2a I117A, S114A I117A Dnmt1 N1580A knock-in murine embryonic stem E14TG2a cells were subcultured every two day cells on 0.15% gelatin-coated dishes in Dulbecco‘s modified Eagle‘s medium (DMEM; Gibco) (15% fetal bovine serum (Gibco), 1× penicillin/streptomycin (Gibco), 0.1 mM sodium pyruvate (Gibco), 0.1 mM 2-mercaptoethanol (Gibco), 1 mM L-alanyl-L-glutamine (Gibco), 1× non-essential amino acids (NEAA; Gibco), 3 µM CHIR99021 (Sigma-Aldrich), 1 µM PD0325901 (Sigma-Aldrich), $1{\times}10^3$ U/ml mouse leukemia inhibitory factor (mLIF; Millipore)). For experiments, cell lines were cultivated in DMEM media supplemented only with mLIF (without CHIR99021 and PD0325901). Cells were maintained at 37 °C in a humidified atmosphere containing 5 % CO_2.

Generation of murine embryonic Mat2A I117A and Mat2A S114A/I117A knock-in cells using CRISPR-Cas9 prime editing

An E14TG2a bi-allelic Dnmt1 N1580A/N1580A cell line [2] bearing a heterozygous I117A or S114A/I117A codon, and E14TG2a Dnmt1 wt bearing heterozygous I117A substitution in exon 4 of the mouse Mat2a locus was generated using genome prime editing PE3 strategy as described previously [4]. pegRNAs and sgRNA were designed using Benchling tool (www.benchling.com). 10 µM of complementary single stranded pegRNA and sgRNA oligonucleotides (Metabion, Table S4) were phosphorylated with 5 U T4 PNK (ThermoFisher Scientific) in PNK buffer supplemented with 1 mM of ATP at 37 °C for 30 min, denatured at 95°C for 5 min and annealed at cooling rate of 0.1 °C /s. pegRNA sequences were cloned into pU6-pegRNA-GG-acceptor (Addgene #132777) incubating 2.5 nM of annealed oligonucleotides, 30 ng of vector, 5 U FastDigest Eco31I (ThermoFisher Scientific) and 5 U T4 DNA ligase (ThermoFisher Scientific) in ligase buffer at 37°C for 5 min followed by incubation at 22 °C for 5 min, repeating the reactions for 6 cycles. sgRNA coding vector was constructed cloning prepared oligonucleotides into phU6-gRNA (Addgene #53188) at BpiI sites following the same ligation conditions. Next, the puromycin coding sequence was amplified from px459 (Addgene #62988) and cloned into pCMV-PE2-P2A-GFP (Addgene #132776) at EcoRI and MssI sites. All plasmids were purified from transformed DH5apha cells using *GeneJet plasmid purification kit* (ThermoFisher Scientific) and verified by Sanger sequencing.
Before cell lipofection, a prime editing vector mixture was prepared in 25 µL of Opti-MEM medium (Gibco) containing 750 ng of PE2, 250 ng of pegRNA and 100 ng of sgRNA plasmids. $7.5{\times}10^4$ E14TG2a cells were transfected with the prime editing vector mixture using Lipofectamine LTX (Invitrogen) according to the manufacturer’s instructions using reverse transfection. After 48 h transfected cells were treated with 2 µg/mL puromycin for another two days. Subsequently, resistant cells were plated for clonal expansion via serial dilution into individual gelatin-coated wells in 96-well plates. After next 6 days of culture, single

clones were picked and passaged in gelatin-coated 24-well plates. Approximately up to two next weeks during clone expansion, 1×10^5 cells of each clone were collected and screened by direct PCR using a Phire Tissue Direct PCR Master Mix and XapI, BspLI or SsiI (Thermo Fisher Scientific) restriction analysis according manufacturer's recommendations to detect Q113N, S114A or I117A events, respectively. All substitutions in the Mat2a coding sequence in selected clones were verified by Sanger sequencing. Primer sequences are listed in Table S3.

Table S3. Primers used for genome prime editing.

**Primer**	**Sequence, 5'..3'**
pegRNA Scaffold Fw	AGAGCTAGAAATAGCAAGTTAAAATAAGGCTAGTCCGTTATCAACTTGAAAAAGTGGCACCGAGTCG
pegRNA Scaffold Rv	GCACCGACTCGGTGCCACTTTTTCAAGTTGATAACGGACTAGCCTTATTTTAACTTGCTATTTCTAG
pegRNA spacer Fw	CACCCCGGTCAAGATGAACACCTTGTTTT
pegRNA spacer Rv	CTCTAAAACAAGGTGTTCATCTTGACCGG
I117A pegRNA RT Fw	GTGCAAATTCACCAGATGCCGCCCAAGGTGTTCATCTTGA
I117A pegRNA RT Rv	AAAATCAAGATGAACACCTTGGGCGGCATCTGGTGAATTT
S114A/I117A pegRNA RT Fw	GTGCGAACAACAGGCACCAGATGCCGCCCAAGGTGTTCATCTTGA
S114A/I117A pegRNA RT Rv	AAAATCAAGATGAACACCTTGGGCGGCATCTGGTGCCTGTTGTTC
sgRNA Fw	CACCGCTGGAACACCTTGTTTTATA
sgRNA Rv	AAACTATAAAACAAGGTGTTCCAGC
Mat2a_PCR Fw	GGAGTTGGCTGCTTGCTTAGTTGTAG
Mat2a_PCR Rv	TCTGGGCGTAACCAAGGCAATGT

Metabolic labeling of mouse embryonic stem cells

For the experiment cells were plated in 24-well plates at a density of 2.5 x 10^4 cells per well and grown for 48 h in DMEM media supplemented with mLIF. Further cells were fed with 0.5, 1, or 2 mM N_3-Met in the DMEM media with 200 µM methionine (Gibco), or in the methionine-deficient medium (Gibco) supplemented with 200 µM L-cysteine (Sigma). Cells were incubated with methionine analog N_3-Met for 3 h, 6 h, 12 h, 18 h, 24 h. Further cells were washed twice with PBS and treated with 0.05% trypsin solution (Gibco) for 1 min at 37 °C. Trypsinization was quenched with DMEM medium, cell suspension was centrifuged (1000 x g, 5 min, 4 °C), and precipitated cells were washed with 200 µL of PBS (1000 x g, 5 min, 4 °C). Cell pellets were stored at -20°C.

Purification of genomic DNA

Collected embryonic stem cells were resuspended in 100 µL PBS and 100 µL of Lysis buffer (0.2 M Tris-HCl, 0.25 M NaCl, 0.025 M EDTA, 0.5% SDS, pH 8) was added, samples were supplemented with 6 U Proteinase K and incubated for 40 min at 56 °C. Further, samples were treated with 0.02 U RNase A (ThermoFisher Scientific) for 10 min at room temperature, genomic DNA was isolated using *Genomic DNA Clean & Concentrator-10 Kit* (Zymo Research) and stored at -20 °C.

Genomic DNA analysis by HPLC-MS/MS

1 µg of modified DNA was digested to nucleotides with 0.01 U/µL of nuclease P1 (Sigma) in 40 µL of P1 reaction buffer (10 mM NaOAc, 1 mM ZnOAc, pH 5.5) for 4 h at 50 °C followed with addition 0.01 U/µL of FastAP alkaline phosphatase (Thermo Fisher Scientific) by an overnight incubation at 37 °C. Next samples were heated at 80 °C for 10 min, and centrifugated (20 000 x *g*, 20 min, 4 °C). Samples were subjected to an integrated HPLC/ESI-MS/MS system (Agilent 1290 Infinity/ 6410 Triple Quad LC/MS) equipped with a Supelco Discovery HS C18 column (7.5 cm × 2.1 mm, 3 µm) by elution with a linear gradient of solvents A (0.0075% formic acid in water) and B (0.0075% formic acid in acetonitrile) at a flow of 0.3 mL/min at 30 °C as follows: 0–5 min, 0% B; 5–15 min, 10% B; 15–20 min, 100% B. Mass spectrometer was operating in the positive ion MRM mode and intensities of nucleoside-specific ion transitions were recorded: N_3-m^5dC 349.2→233.1, m^5dC 242.1→126.1; dC 228.1→112.0, dG 268.1→152.1. Signals were calibrated using FastAP-treated nucleotides for standard curves as previously described [2]. All signals were normalized to dG. Data were analyzed using Agilent MassHunter software and Microsoft Excel.

Cell viability MTT assay

Embryonic stem cells were seeded into 96-well plates at a density of 5 x 10^3 cells per well 48 h before the experiment, incubated with 0.5, 1, or 2 mM N_3-Met in the DMEM media with 200 µM methionine, or in the methionine-deficient medium supplemented with 200 µM L-cysteine for 24 h. Next day, medium was removed and cells incubated with 500 µL of 0.1 mg/mL 3-(4,5-dimethylthiazol-2-yl)-2,5-diphenyltetrazolium bromide (MTT, Sigma) in Opti-MEM medium for 1 h at 37 °C, washed twice with 200 µL PBS and lysed with 200 µL isopropanol. Formazan absorption in samples was measured at 570 nm using a Synergy H4 (Biotek) plate reader.

RNA isolation

Total RNA from cell lines samples was isolated using *GeneJET RNA purification kit* (ThermoFisher Scientific) according to manufacturer's instructions. RNA samples were eluted in pre-heated nuclease-free water, stored at -80 °C until further analysis. Immediately before use, isolated RNA was treated with dsDNase (ThermoFisher Scientific) according to manufacturer's instructions.

Quantitative real-time PCR analysis

For the *Mat2a* and *Dnmt1* mRNA expression analysis 1 µg of total RNA previously treated with dsDNase were used for the cDNA synthesis. cDNA was prepared in 20 µl of the RT reaction in the RT buffer which contained 200 units of RevertAid Reverse Transcriptase, 1 mM dNTP, 0.16 µg Random hexamer mixture, 16 units of RNase inhibitor RiboLock (ThermoFisher Scientific). The mixture was incubated for 20 min at 25 °C and for 60 min at 42 °C and then heat-inactivated for 10 min at 70 °C. Real-time PCR was performed with SYBR Green PCR master mix (ThermoFisher Scientific) according to the manufacturer's instructions. Relative quantification of changes of gene expression levels was performed using the comparative

C_t (threshold cycle) method. Endogenous control *Gapdh* was used as endogenous control for the mRNA expression normalization. Primers (Metabion) are listed in Supplementary Table S5. QPCR analysis was conducted on Rotor-Gene 6000 (Corbett Life Science) equipment. PCR products using wt, I117A, S114A I117A allele-specific primers (depicted as sp- primers in Table S4) were separated on a 2% agarose gel.

Table S4. Primers used for QPCR.

Primer	Sequence 5'..3'
Mat2a FW	GCTTCACGAGGCGTTCAT
Mat2a REV	AGCATCACTGATTTGGTCAC
Dnmt1 FW	AAGAATGGTGTTGTCTACCGAC
Dnmt1 REV	CATCCAGGTTGCTCCCCTTG
GapdhFW	AGGTCGGTGTGAACGGATTTG
Gapdh REV	TGTAGACCATGTAGTTGAGGTCA
Mat2a sp-I117A FW	CTTGGAACAACAGTCACCAGATGCC
Mat2a sp-S114A I117A FW	GTTGGTTGCCTTGGAACAACAGG
Mat2a sp-wt FW	GTTGGTTGCCTTGGAACAACAGT
Mat2a sp-REV	GGCAATGTACCATTGCGGCGTAG

Western blotting

Cells were cultivated in 6-well plates, washed with PBS and scraped into 1.5 ml tubes, next lysed for 30 min on ice in 50 µl RIPA buffer: 50 mM Tris-HCl (pH 7.4), 150 mM NaCl, 1 % Triton X-100, 0.1% sodium deoxycholate, 10 mM KF, 1 mM Na_3VO_4, 1 mM PMSF and *cOmplete*™ EDTA-free Protease Inhibitor Cocktail (Roche) (one tablet to 10 ml buffer). Then lysates were centrifuged for 15 min at 20 000 × *g* at 4 °C. Supernatants were collected and protein concentration was determined by the BCA protein assay (ThermoScientific). 10 µg of protein samples were subjected to 12% SDS-PAGE and then transferred to PVDF membrane (Milipore) by semidry blotting. The membranes were blocked with RotiBlock (Roth) in PBS-T buffer for 1 h at 4 °C. Next, membranes were incubated with anti-MAT2A (NB110-94158, NovusBio) and with anti-DNMT1 (ab87654, Abcam) at 1:1000 dilution, overnight in PBS-T buffer at 4 °C. Membrane-bound primary antibodies were detected by horseradish peroxidase-conjugated secondary anti-rabbit IgG antibody (31460, Pierce). Secondary antibodies were diluted at 1:10000 in PBS-T, and membranes were incubated for 2 h at 4 °C. Antibody binding was visualized using SuperSignal West Pico PLUS Chemiluminescent Substrate, (Thermo Scientific) and visualized with *ChemiDoc*™ imaging system. Next, membrane was incubated with stripping buffer (62.5 mM Tris-HCl (pH 6.7), 2% SDS, 100 mM 2-mercaptoethanol) for 30 min at 50 °C, and washed 3 times with PBS-T, next blocked and incubated with anti-GAPDH (sc32233, SantaCruz) at dilution 1:5000 in PBS-T. In this case, primary antibody was detected by horseradish peroxidase-conjugated secondary anti-mouse IgG antibody (31430, Pierce).

Preparation of Dnmt-TOP-seq libraries of mESCs

The derived *Mat2a* $^{wt}/^{I117A}$ *Dnmt1* $^{N1580A}/^{N1580A}$ (I117A) cells and control E14TG2a *Mat2a*$^{wt/wt}$ *Dnmt1*$^{wt/wt}$ cells (wt) were seeded in 24-well plates at a density of 2.5 x 10^4 cells per well and grown for 48 h in DMEM media supplemented with mLIF. Further cells were fed with 2 mM N_3-Met in a DMEM medium containing 200 µM methionine for 24 h. Preparation of Dnmt-TOP-seq libraries and processing of sequencing data were carried out as described.[2] NGS data have been deposited with the GEO database (Accession No. GSE267304).

Analysis of non-specific protein labeling

The derived *Mat2a* $^{wt}/^{I117A}$ *Dnmt1* $^{N1580A}/^{N1580A}$ (I117A) cells and control E14TG2a *Mat2a*$^{wt/wt}$ *Dnmt1*$^{wt/wt}$ cells (wt) were preincubated for 48 h in 6-well plates at a density of 12.5 x 10^4 cells per well and then incubated for 24 h with 1 mM N_3-Met in DMEM medium with 200 µM or no Met and 200 µM L-cysteine. Cells were lysed in a 100 µl ice-cold RIPA buffer containing a 1 × Complete EDTA-free protease inhibitor cocktail. Then lysates were centrifuged for 15 min at 20 000 × *g* at 4 °C and protein concentration was determined by the BCA protein assay. Whole cell lysates (100 µl, 150–200 µg total protein) were precipitated with a mixture of 400 µL ice-cold methanol, 100 µL chloroform and 300 µL water and centrifuged at 14 000 *g* for 5 min. Pellet was washed with 400 µL of methanol, air-dried for 15 min and dissolved in 100 µL of 50 mM TEA, pH 7.4, 150 mM NaCl and 4% SDS, containing a 1 × Complete EDTA-free protease inhibitor cocktail. 25 µL aliquots were was subjected to Cy5 labeling by adding 100 µM DBCO-Cy5 and incubating at 37 °C for 1.5 h. Excess dye was removed by protein precipitation as described above, and pellets were dissolved in 50 µL 1x gel loading buffer (40 mM Tris-HCl (pH 7.4), 10 mM EDTA, 4% SDS, 10% glycerol, 10% 2-mercaptoethanol). Samples were heated at 95 °C and 5–7.5 µL aliquots were loaded for analysis by 12% SDS-PAGE. Gel was fixed in a gel fixing buffer (40% methanol, 10% acetic acid) for at least 1 h. Fluorescent bands were visualized by in-gel Cy5 fluorescence (λ_{ex}=649 nm, λ_{em}= 666 nm) scanning with a *ChemiDoc*^{TM} imaging system, followed by Coomassie Brilliant Blue dye (0,09% Brillant Blue R 250, 45% ethanol, 9% acetic acid) staining.

CHEMICAL SYNTHESIS of *S*-(6-azidohex-2-yn-1-yl)-L-homocysteine

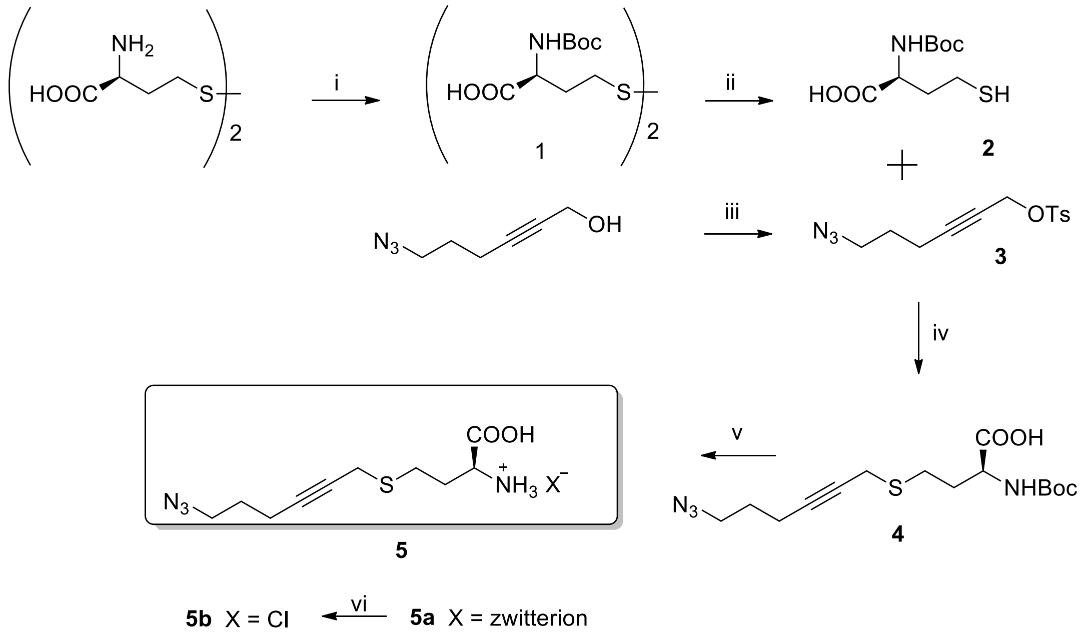

Scheme S1. *Reagents and conditions*: i = K_2CO_3, Boc_2O, H_2O, Dioxane, 0 °C, then rt 24 h; ii = TCEP*HCl, DMF, H_2O, rt, Ar; iii = TosCl, $(CH_3)_3COK$, THF, 0 °C, then rt, Ar; iv = $(CH_3)_3COK$, THF, - 50 °C, Ar, 2h, then warmed to - 20 °C ; v = a) CH_2Cl_2, F_3CCOOH, 0 °C, Ar, 15 min; b) rt, N_2 stream; vi = conc. HCl.

General information

^1H NMR and ^{13}C NMR spectra were recorded on Bruker 400 (400 MHz and 100 MHz, respectively) using residual solvent signals as internal standard. All reactions and purity of the synthesized compounds were monitored by TLC using Silica gel 60 F_{254} aluminium sheets (Merck). Visualization was accomplished by UV light or with potassium permanganate solution. Merck Silica gel 60 (230–400 mesh) was used for column chromatography. Melting points were determined in closed capillaries with a digital melting point IA9100 series apparatus (Thermo Fischer Scientific) and are uncorrected. IR spectra were run on Agilent Cary 630 FTIR spectrophotometer with a diamond ATR module. HRMS data were obtained using an Agilent 6230 TOF mass spectrometer (ESI).

The starting compounds were synthesized according to known methods (6-azidohex-2-yn-1-ol) [1] or commercially available.

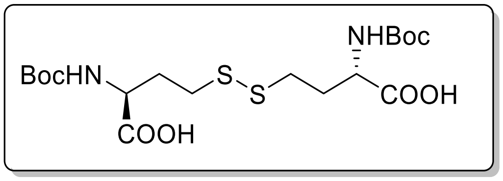

***N*,*N*'-di(*tert*-butoxycarbonyl)-L-homocystine (1).**

L-homocystine (1.12 g, 4.19 mmol, 1 equiv.) was dissolved in 35 mL of 1 M K_2CO_3 aqueous solution. Then, di-*tert*-butyl dicarbonate (Boc_2O) (1.97 g; 9.01 mmol, 2.2 equiv.) in 32 mL of dioxane was added drop-wise at 0 °C over 30 min. and stirred for another 1 h at 0 °C temperature. Then the mixture was left at ambient temperature for 48 hours. The reaction volume was reduced to 1/3 by distillation under reduced pressure, and the acidity of the aqueous solution was adjusted to pH ≈ 2–3 by 5% citric acid under ice bath cooling. The emulsion was extracted with ethyl acetate (3×75 mL). The combined organic layers were washed with water (2×20 mL), with brine (2×25 mL) and dried over Na_2SO_4. Then filtered, and ethyl acetate was removed by distillation under reduced pressure. The residue was dried in a vacuum for 2 hours.

White crystals (1.89 g, 96%), mp 151–152 °C. **^{1}H NMR** (400 MHz, DMSO-*d*$_6$) *δ* (ppm): 1.38 (s, 18H, C(C**H$_3$**)$_3$), 1.79-1.95 (m, 2H, SCH_2C**H$_a$**CH_b), 1.96-2.12 (m, 2H, SCH_2CH_aC**H$_b$**), 2.63-2.83 (m, 4H, SC**H$_2$**), 3.86-4.11 (m, 2H, C**H**NHBoc), 7.18 (d, ^3J = 8.1 Hz, 2H, N**H**Boc), 12.60 (br s, 2H, COO**H**). **^{13}C NMR** (100 MHz, DMSO-*d*$_6$) *δ* (ppm): 28.2, 30.3, 34.2, 52.2, 78.2, 155.6, 173.8.

NMR spectra in CD_3OD and a melting point of compound **1** are described in ref [5].

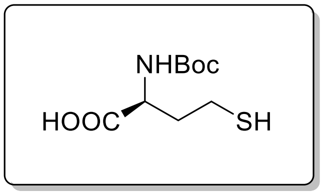

***N*-*tert*-butoxycarbonyl-L-homocysteine (2).**

To a solution of *N*-di-*tert*-butoxycarbonyl-L-homocystine **1** (150 mg, 0.32 mmol, 1 equiv.) in 3.33 mL of DMF solution of tris(2-carboxyethyl)phosphine hydrochloride (TCEP·HCl) (187.2 mg, 0.64 mmol, 2 equiv.) in 0.5 mL milliQ H_2O was added. The reaction mixture was stirred at ambient temperature under an argon atmosphere and monitored by thin-layer chromatography using CH_2Cl_2/EtOH/AcOH 1:1:0.01 as eluent. After the reaction was completed (2–3 h), the mixture was cooled in an ice bath and diluted with 15 ml of 1.5 M $Na_2S_2O_5$ solution, then stirred for another 15 min under cooling. The product was extracted with ethyl acetate (1×50 mL, then 2 × 15 mL). The combined organic layers were washed with water (20 mL), brine (20 mL) and dried over Na_2SO_4. Then filtered, and ethyl acetate was removed by distillation under reduced pressure. The product was dried in a vacuum and stored cold (- 20 °C) and under an argon atmosphere.

Oil (103 mg, 67%). **ATR-FTIR**: 3330 cm^{-1} (NH), 2562 cm^{-1} (SH), 1701 cm^{-1}, 1647 cm^{-1} (C=O), 1159 cm^{-1} (C-O). **^{1}H NMR** (400 MHz, $CDCl_3$) δ (ppm): 1.44 (s, 9H, C(C**H$_3$**)$_3$), 1.57 (t, ^3J = 8.1 Hz, 1H, S**H**), 1.91-2.04 (m, 1H,

$SCH_2CH_aH_b$), 2.11-2.22 (m, 1H, $SCH_2CH_a\mathbf{H_b}$), 2.45-2.78 (m, 2H, $S\mathbf{CH_2}$), 4.26-4.60 (m, 1H, NHC**H**), 5.13 and 6.66 (d, ^3J = 8,5 Hz, 1H, N**H**), 10.82 (br s, 1H, COO**H**). **^{13}C NMR** (100 MHz, $CDCl_3$) δ (ppm): 20.9, 28.4, 37.0, 52.4, 80.6, 155.7, 177.6.

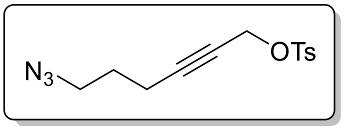

6-azidohex-2-yn-1-yl tosylate (3)

To a solution of 6-azidohex-2-yn-1-ol (0.40 g, 2.87 mmol, 1 equiv.) in 14 mL of anhydrous THF potassium *tert*-butoxide (0.29 g, 2.59 mmol, 0.9 equiv.) was added at 0 °C under argon atmosphere. Stirring for several minutes gave clear solution, then tosyl chloride (0.49 g, 2.59 mmol, 0.9 equiv.) was added. The reaction mixture was stirred for 2 h, and then the solvent was removed by distillation under reduced pressure. The residue was purified by column chromatography using toluene as an eluent (R_f = 0.3).

Clear oil (0.58 g, 69%). **ATR-FTIR**: 2239 cm^{-1} (C≡C), 2096 cm^{-1} (N_3), 1364 cm^{-1} (O=S=O) ν_{as}, 1174 cm^{-1} (O=S=O) ν_s. **^{1}H NMR** (400 MHz, $CDCl_3$) δ (ppm): 1.65 (qu, ^3J = 6.8 Hz, 2H, $CH_2\mathbf{CH_2}CH_2$), 2.21 (tt, ^3J = 6.8 Hz, ^5J = 2.4 Hz, 2H, $CH_2\mathbf{CH_2}CH_2$), 2.45 (s, 3H, CH_3), 3.30 (t, ^3J = 6.8 Hz, 2H, $N_3\mathbf{CH_2}$), 4.68 (t, ^5J = 2.4 Hz, 2H, $\mathbf{CH_2}O$), 7.35 (d, ^3J = 8.2 Hz, 2H, Ar**H**), 7.81 (d, ^3J = 8.2 Hz, 2H, Ar**H**). **^{13}C NMR** (100 MHz, $CDCl_3$) δ (ppm): 16.1, 21.8, 27.4, 50.1, 58.5, 73.2, 88.6, 128.2, 129.9, 133.4, 145.1.

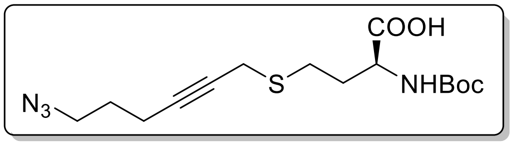

S-(6-azidohex-2-yn-1-yl)-*N*-(*tert*-butoxycarbonyl)-L-homocysteine (4).

To a solution of *N*-*tert*-butoxycarbonyl-L-homocysteine **2** (103 mg, 0.438 mmol, 1 equiv.) in 7 mL of anhydrous THF potassium *tert*-butoxide (95.8 mg, 0.854 mmol, 1.95 equiv.) was added at -50 °C under argon atmosphere. After stirring for 15 min, 6-azidohex-2-yn-1-yl tosylate **3** (122.6 mg, 0.416 mmol, 0.95 equiv.) was added at the same temperature and the reaction mixture was stirred for 2 h. Then the reaction mixture was warmed to -20 °C and 5% citric acid solution (30 mL) was added to it, resulting solution stirred for 15 min. The product was extracted with ethyl acetate (2×30 mL). The combined organic layers were washed with water (20 mL), and brine (20 mL) and dried over Na_2SO_4. Then filtered, and ethyl acetate was removed by distillation under reduced pressure. The residue was purified by column chromatography using EtOAc/CH_2Cl_2/AcOH (5:3:0.04) as an eluent (R_f = 0.45).

Yellowish oil (84.3 mg, 54%). **ATR-FTIR**: 3325 cm^{-1} (NH), 2095 cm^{-1} (N_3), 1709, 1654 cm^{-1} (C=O), 1158 cm^{-1} (C-O). **^{1}H NMR** (400 MHz, $CDCl_3$) δ (ppm): 1.45 (s, 9H, $C(\mathbf{CH_3})_3$), 1.77 (qu, ^3J = 6.7 Hz, 2H, $CH_2\mathbf{CH_2}CH_2$), 1.92-2.05 (m, 1H, $SCH_2\mathbf{CH_a}CH_b$), 2.13-2.27 (m, 1H, $SCH_2CH_a\mathbf{CH_b}$), 2.32 (tt, ^3J = 6.7 Hz, ^5J = 2.4 Hz, 2H, $N_3CH_2CH_2\mathbf{CH_2}$), 2.75 (t, ^3J = 7.6 Hz, 2H, $S\mathbf{CH_2}CH_2$), 3.26, (t, ^5J = 2.4 Hz, 2H, $S\mathbf{CH_2}$C≡C), 3.40 (t, ^3J = 6.7 Hz, 2H,

N_3CH_2), 4.26-4.51 (m, 1H, NHC**H**), 5.14 and 6.43 (d, ^3J = 8.0 Hz, 1H, N**H**), 8.85 (br s, 1H, COO**H**). **^{13}C NMR** (100 MHz, $CDCl_3$) δ (ppm): 16.2, 19.8, 27.5, 28.0, 28.4, 31.9, 50.3, 52.8, 76.7, 80.6, 82.0, 155.8, 177.2.

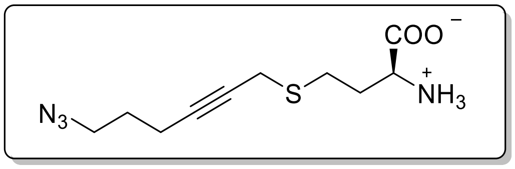

S-(6-azidohex-2-yn-1-yl)-L-homocysteine zwitterion (5a).

S-(6-Azidohex-2-yn-1-yl)-*N*-(*tert*-butoxycarbonyl)-L-homocysteine **4** (50 mg, 0.14 mmol) was dissolved in 2 mL CH_2Cl_2/TFA 1:1 (v/v) solution at 0 °C under argon atmosphere and stirred at ambient temperature for 30 minutes. After completion of the reaction, the solvent mixture was removed under a nitrogen stream, and residue was purged by nitrogen stream until pH of this stream was not acidic anymore (wet universal paper indicators were used for detection). Then the residue was dissolved in 5 mL miliQ H_2O and washed with CH_2Cl_2 (5×5 mL). The aqueous layer was removed by distillation under reduced pressure, residue was dissolved in 1 mL of MeOH and 5 mL of Et_2O was added. The resulting cloudy mixture was centrifuged, liquid decanted and residue dried in a vacuum.

White solid (22.5 mg, 63% - prepared from 50 mg of **4**), dec. 140 °C. **ATR-FTIR**: 2094 cm^{-1} (N_3), 1560cm^{-1} (COO^-) $ν_{as}$, 1404 cm^{-1} (COO^-) $ν_{s.}$

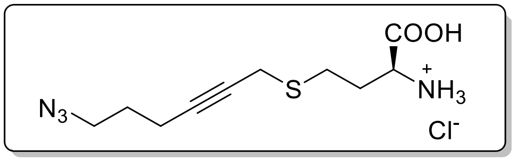

S-(6-azidohex-2-yn-1-yl)-L-homocysteine chloride (5b).

S-(6-azidohex-2-yn-1-yl)-L-homocysteine zwitterion **5a** (22.5 mg, 0.088 mmol) was dissolved in 1 ml of conc. HCl and the solvent was removed under a water jet pump vacuum.

White crystals (25 mg, 97%), dec. 105 °C. **ATR-FTIR**: 2087 cm^{-1} (N_3), 1711 cm^{-1} ($C{=}O^-$). **^{1}H NMR** (400 MHz, D_2O) δ (ppm): 1.64 (qu, ^3J = 6.8 Hz, 2H, $CH_2C\mathbf{H_2}CH_2$), 2.02-2.12 (m, 1H, $SCH_2C\mathbf{H_a}CH_b$), 2.15-2.27 (m, 3H, $SCH_2CH_aC\mathbf{H_b}$ + $CH_2C\mathbf{H_2}C{\equiv}C$), 2.73 (t, ^3J = 7.7 Hz, 2H, $SC\mathbf{H_2}CH_2$), 3.24 (t, ^5J = 2.4 Hz, 2H, $SC\mathbf{H_2}C{\equiv}C$), 3.01 (t, ^3J = 6.8 Hz, 2H, $N_3C\mathbf{H_2}$), 4.04 (t, ^3J = 6.4 Hz, 1H, NH_3C**H**). **^{13}C NMR** (100 MHz, D_2O) δ (ppm): 15.4, 18.5, 26.6, 27.0, 29.2, 50.1, 52.1, 76.8, 83.2, 172.1. HRMS: m/z $[M+H]^+$calcd for $C_{10}H_{17}N_4O_2S$: 257.1067; found: 257.105

SUPPORTING REFERENCES

(1) Lukinavičius, G.; Tomkuvienė, M.; Masevičius, V.; Klimašauskas, S.: Enhanced Chemical Stability of AdoMet Analogues for Improved Methyltransferase-Directed Labeling of DNA. *ACS Chem Biol* **2013**, *8*, 1134-1139.

(2) Stankevičius, V.; Gibas, P.; Masiulionytė, B.; Gasiulė, L.; Masevičius, V.; Klimašauskas, S.; Vilkaitis, G.: Selective chemical tracking of Dnmt1 catalytic activity in live cells. *Mol Cell* **2022**, *82*, 1053-1065. e8.

(3) Gerasimaitė, R.; Vilkaitis, G.; Klimašauskas, S.: A directed evolution design of a GCG-specific DNA hemimethylase. *Nucleic Acids Res* **2009**, *37*, 7332-7341.

(4) Anzalone, A. V.; Randolph, P. B.; Davis, J. R.; Sousa, A. A.; Koblan, L. W.; Levy, J. M.; Chen, P. J.; Wilson, C.; Newby, G. A.; Raguram, A.; Liu, D. R.: Search-and-replace genome editing without double-strand breaks or donor DNA. *Nature* **2019**, *576*, 149-157.

(5) Bourdier, T.; Fookes, C. J. R.; Pham, T. Q.; Greguric, I.; Katsifis, A.: Synthesis and stability of *S*-(2-[^{18}F]fluoroethyl)-L-homocysteine for potential tumour imaging. *J Label Compd Radiopharm* **2008**, *51*, 369-373.

^{1}H and ^{13}C NMR and IR spectra of the synthesized compounds

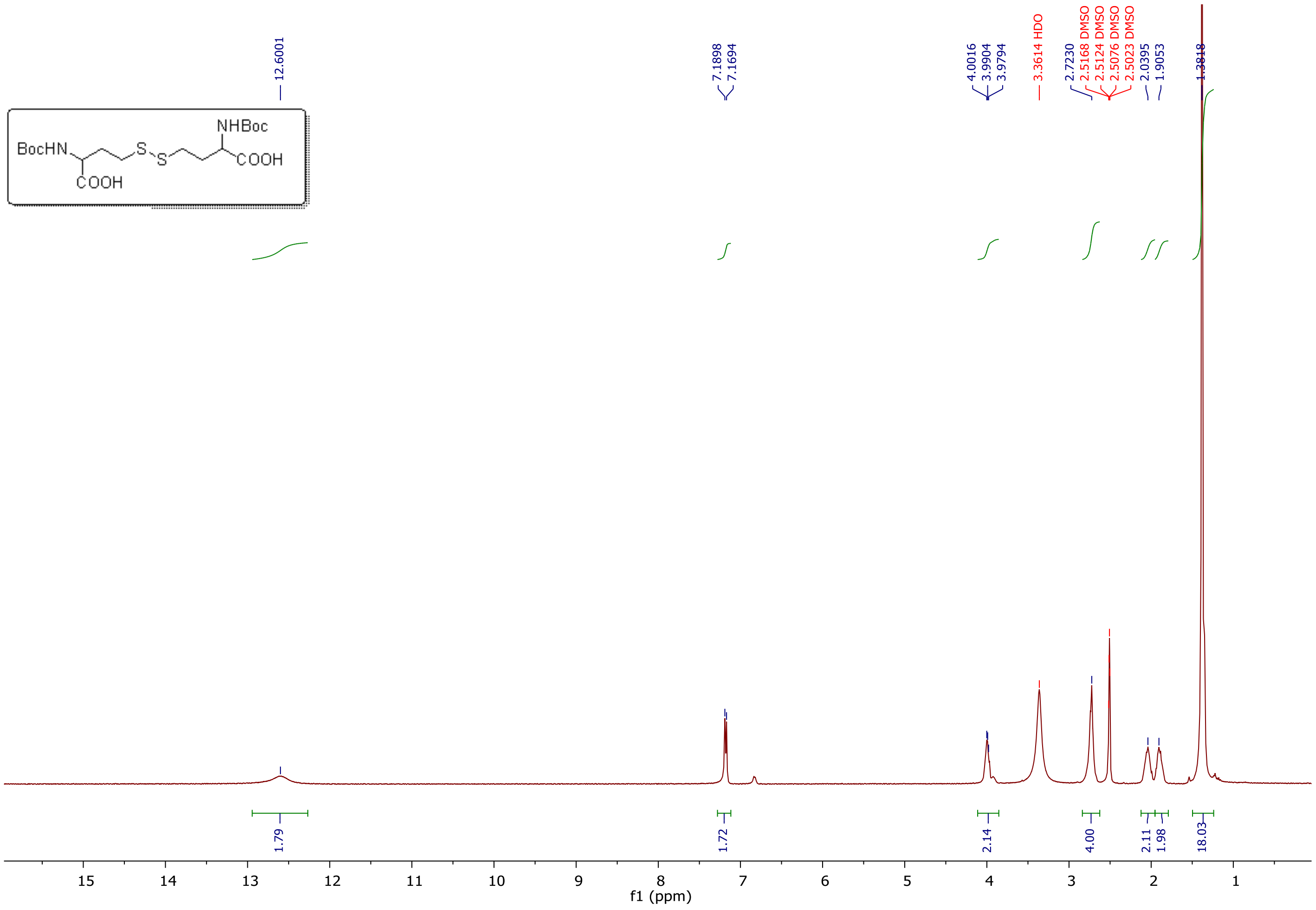

^{1}H NMR spectrum of *N*,*N'*-di(*tert*-butoxycarbonyl)-L-homocystine (1)

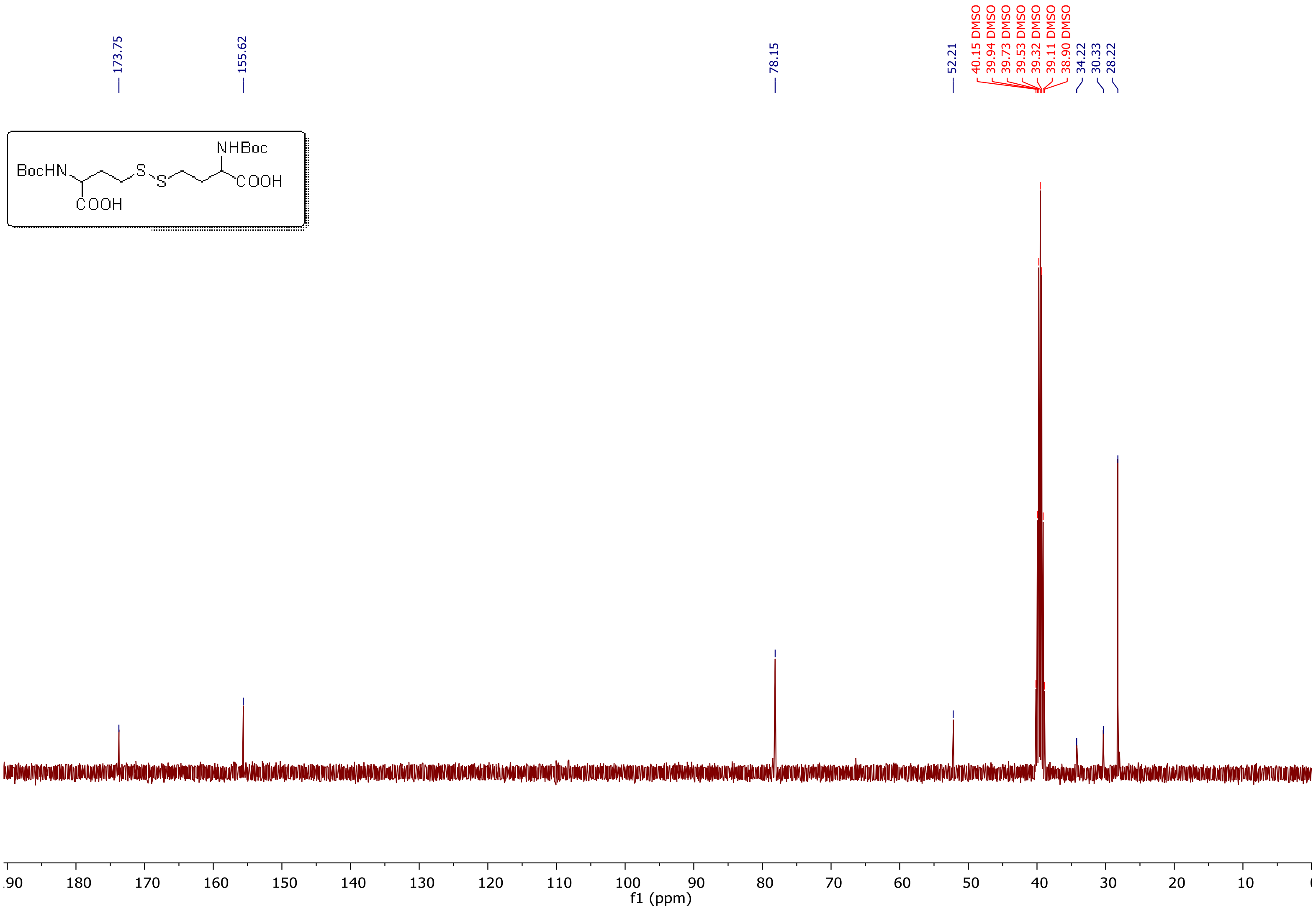

^{13}C NMR spectrum of *N*,*N'*-di(*tert*-butoxycarbonyl)-L-homocystine (1)

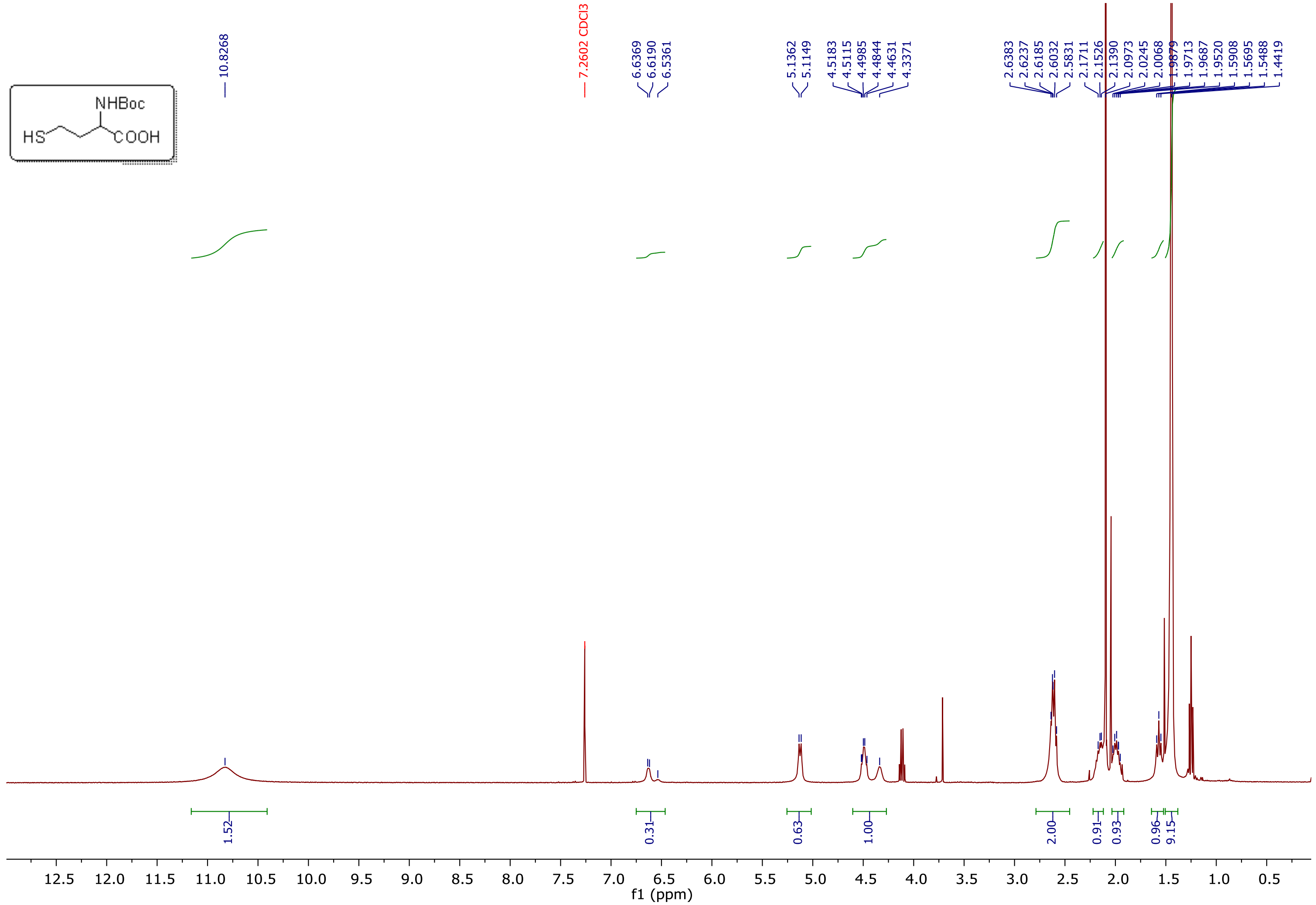

Residual solvent signals: EtOAc 1.25, 2.04, 4.12; Dioxane 3.71; AcOH 2.10, 10.83; water 1.52.

^{1}H NMR spectrum of *N*-*tert*-butoxycarbonyl-L-homocysteine (2)

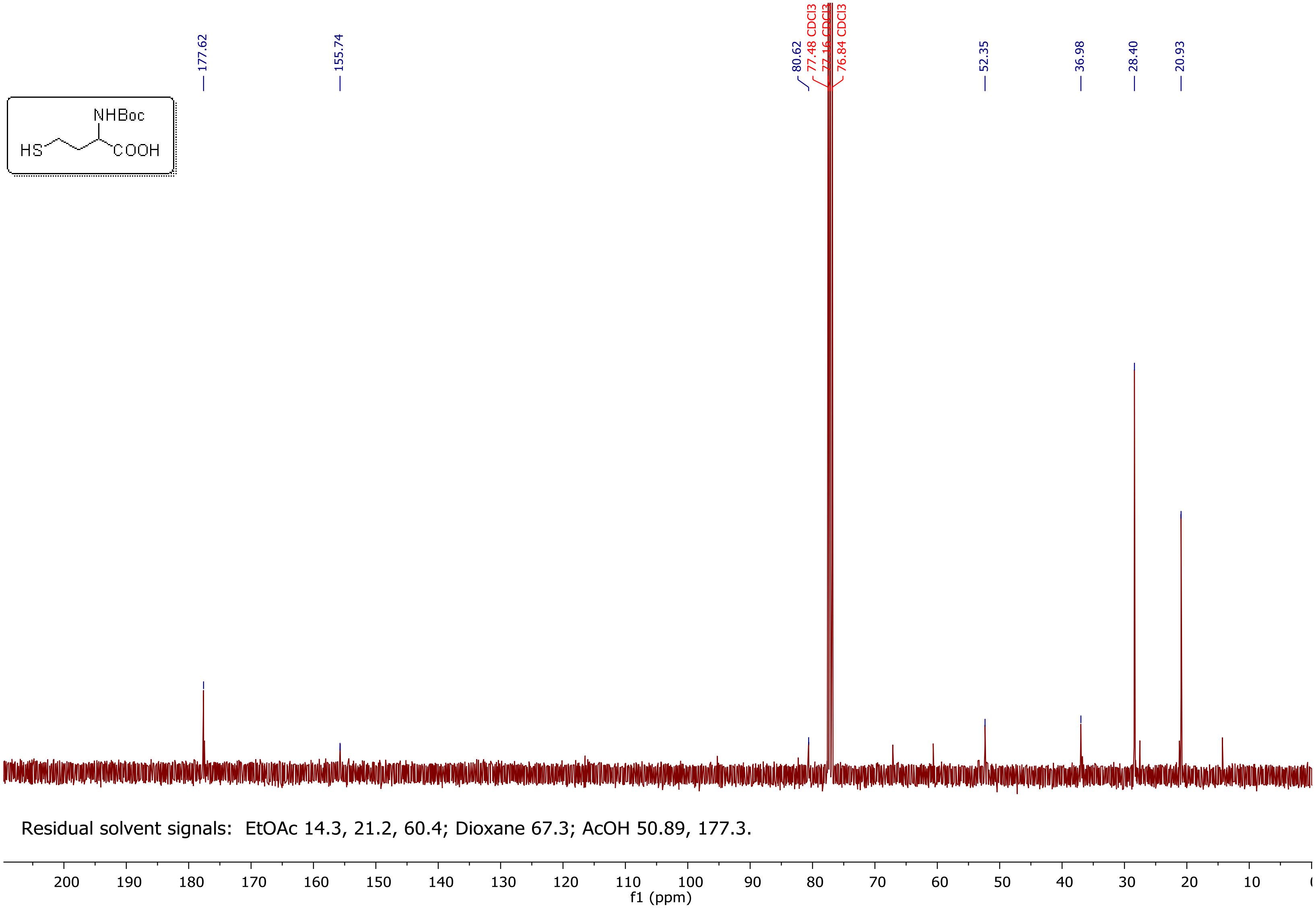

^{13}C NMR spectrum of *N*-*tert*-butoxycarbonyl-L-homocysteine (2)

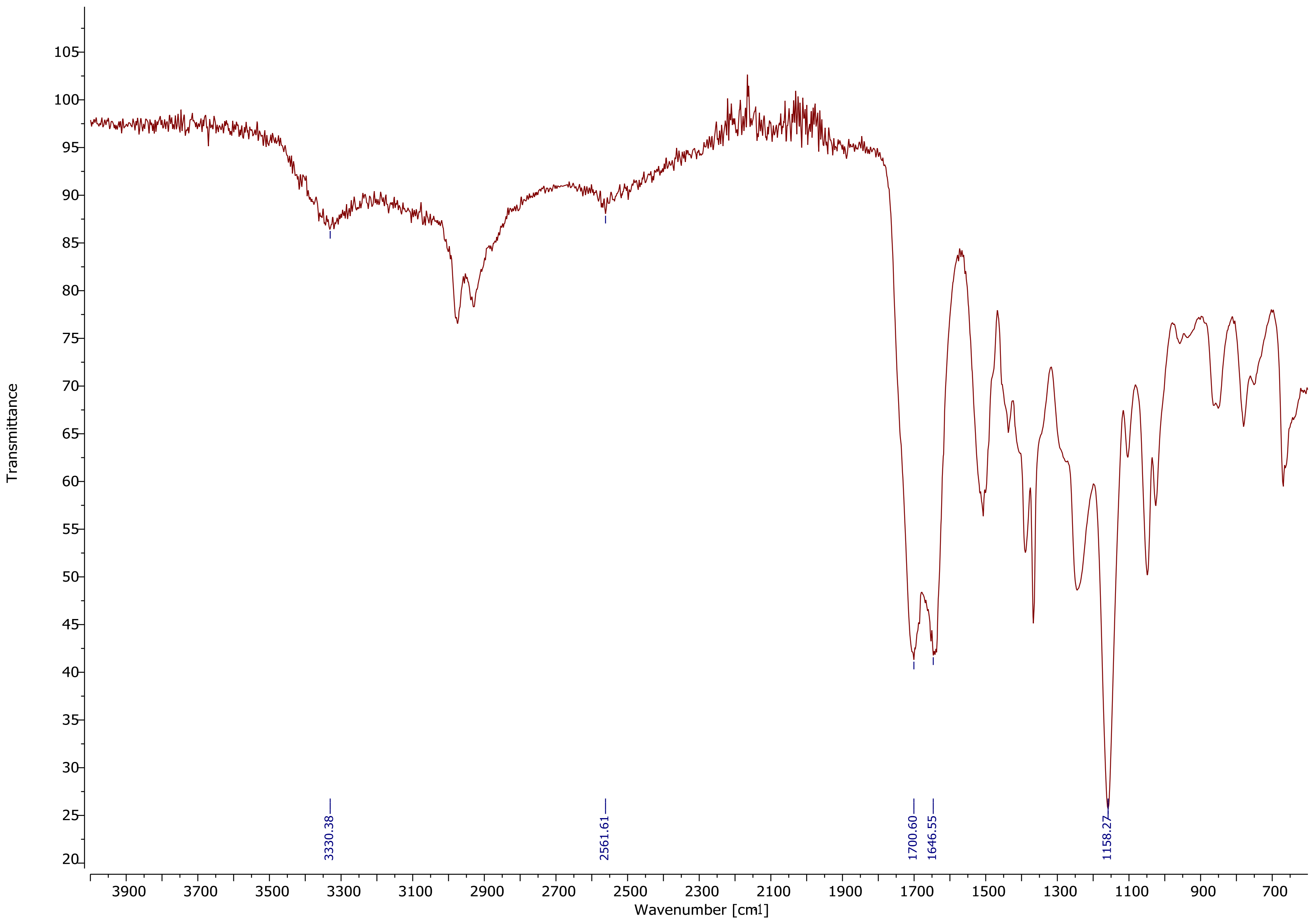

IR spectrum of N-tert-butoxycarbonyl-L-homocysteine (2)

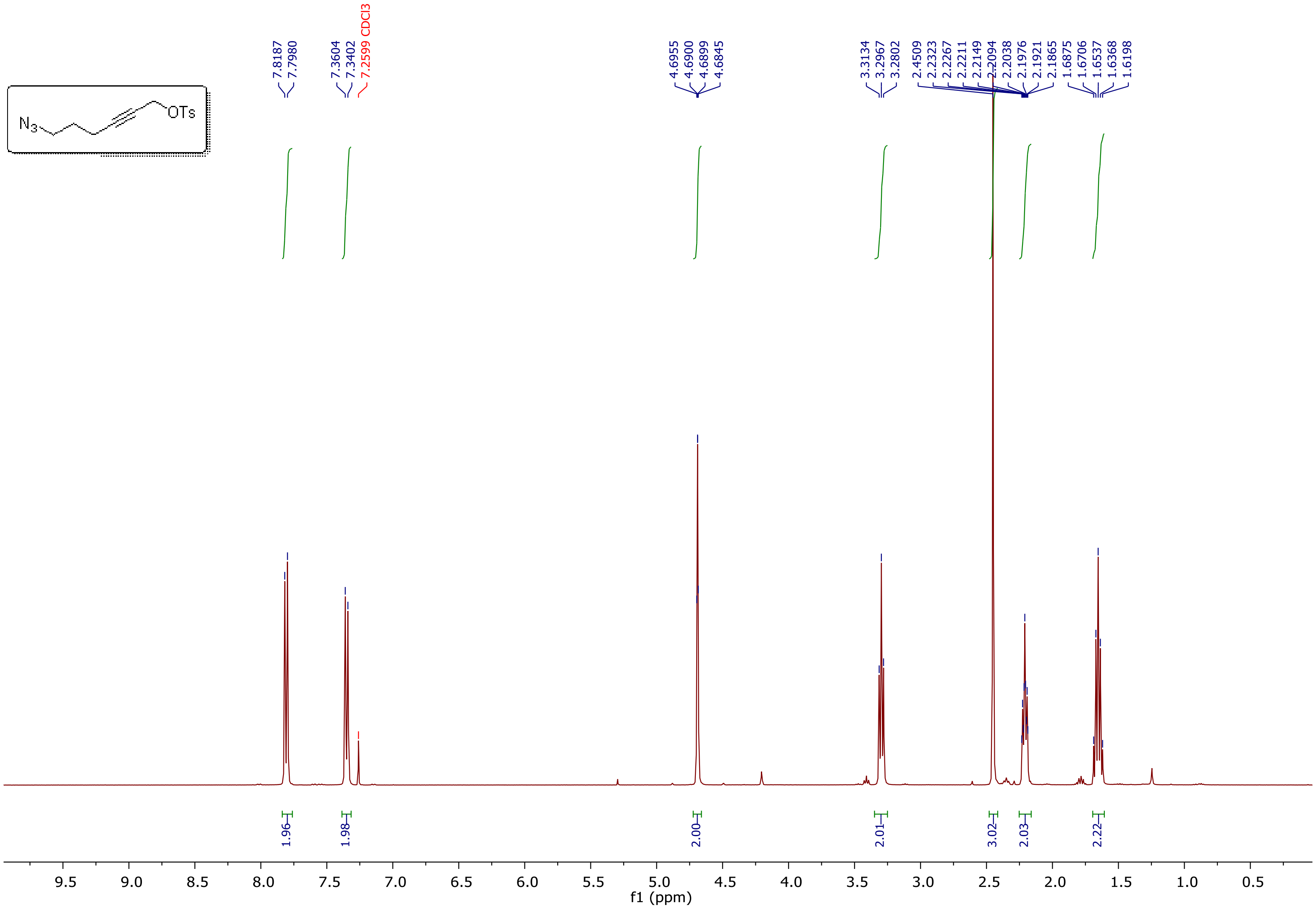

^{1}H NMR spectrum of 6-azidohex-2-yn-1-yl 4-tosylate (3)

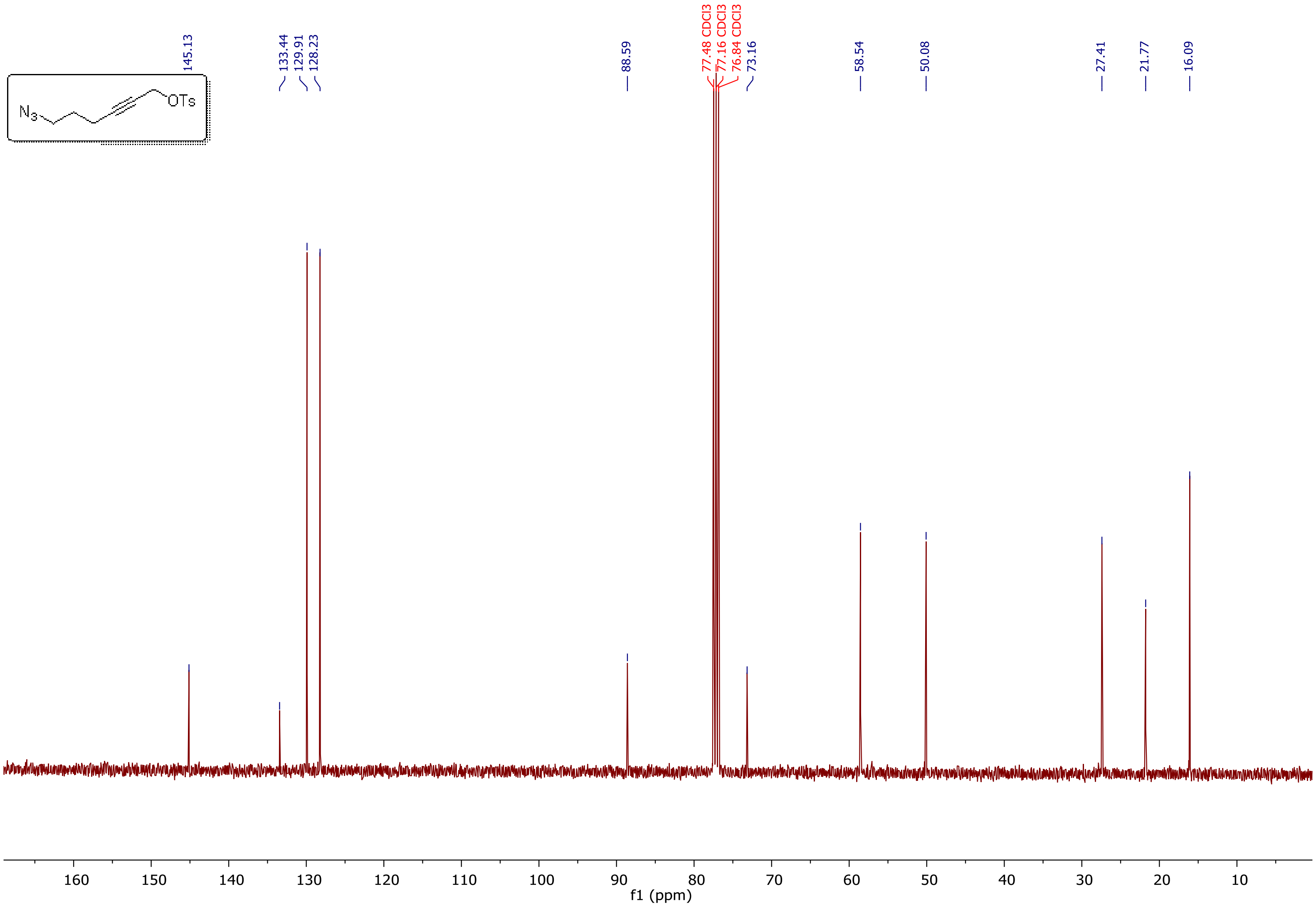

^{13}C NMR spectrum of 6-azidohex-2-yn-1-yl 4-tosylate (3)

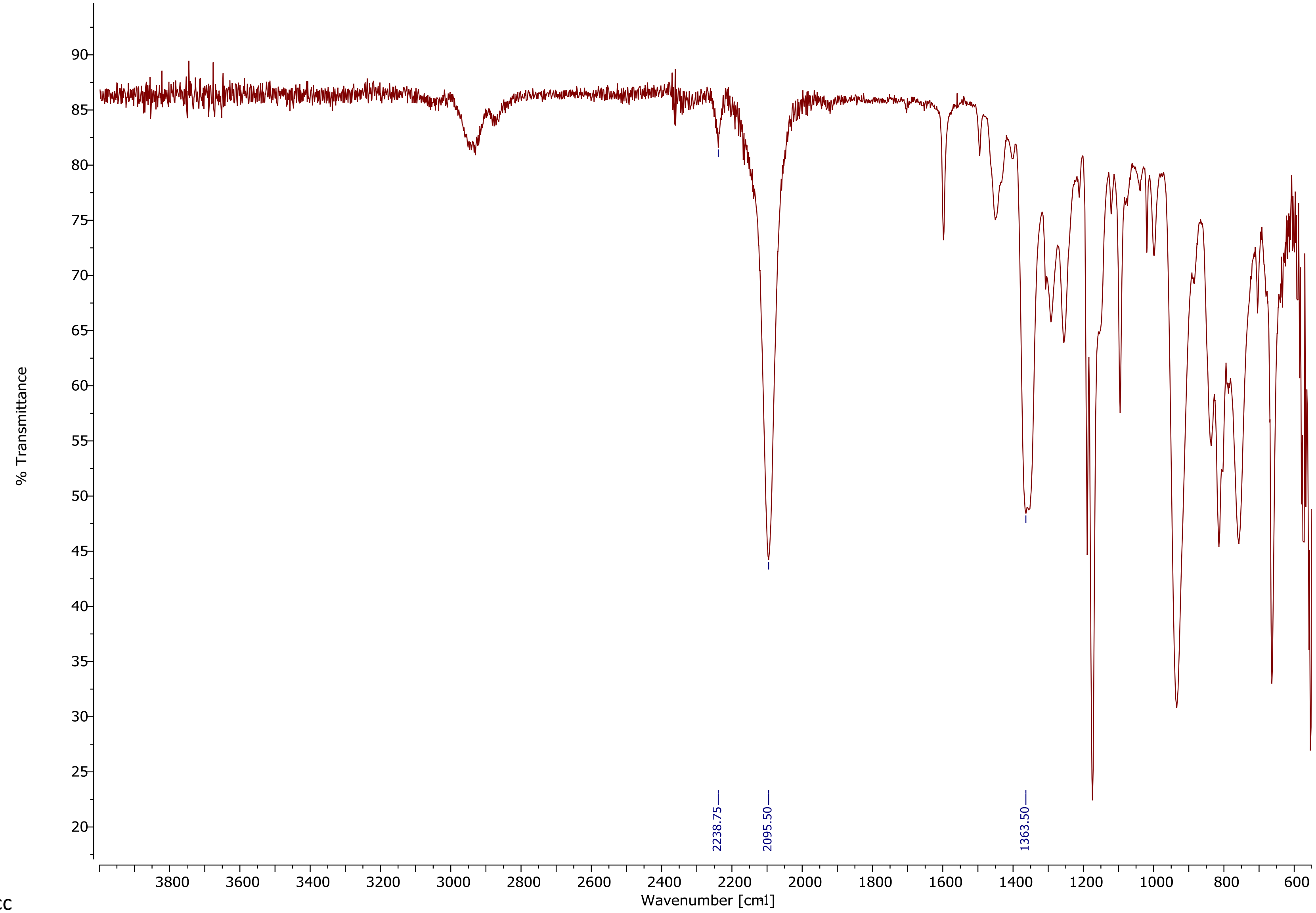

cc

IR spectrum of 6-azidohex-2-yn-1-yl 4-tosylate (3)

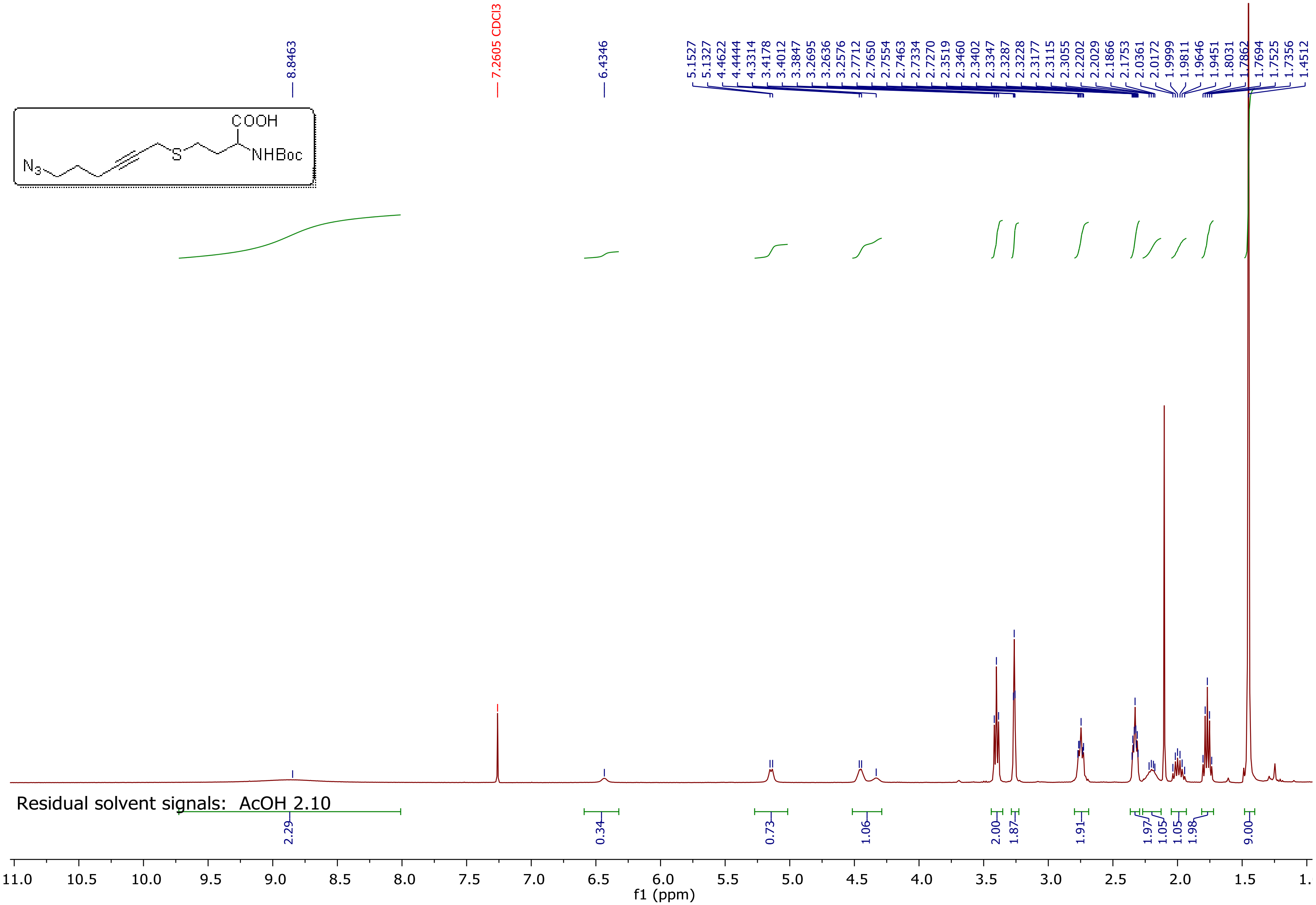

^{1}H NMR spectrum of S-(6-Azidohex-2-yn-1-yl)-*N*-(*tert*-butoxycarbonyl)-L-homocysteine (4)

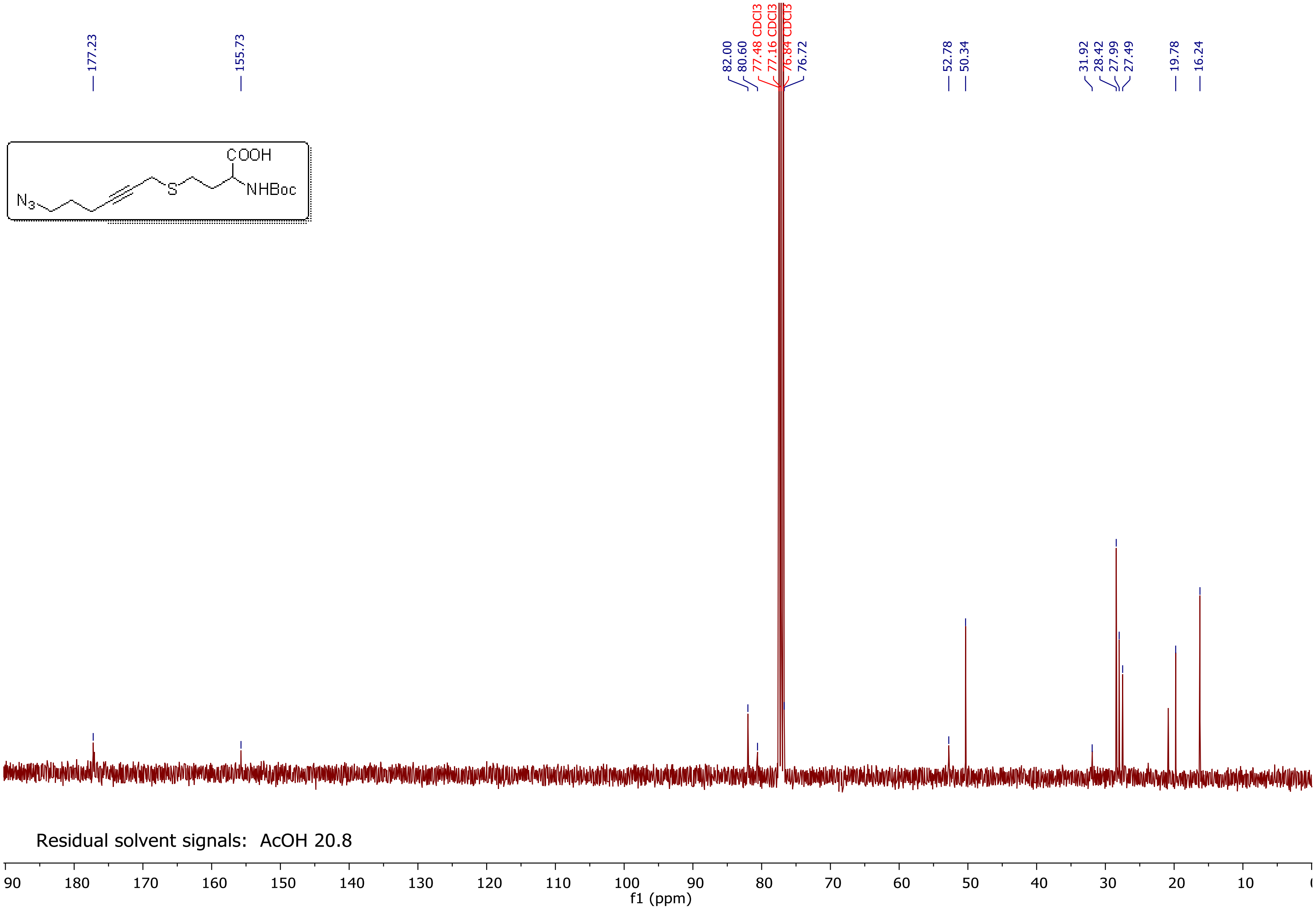

^{13}C NMR spectrum of S-(6-Azidohex-2-yn-1-yl)-*N*-(*tert*-butoxycarbonyl)-L-homocysteine (4)

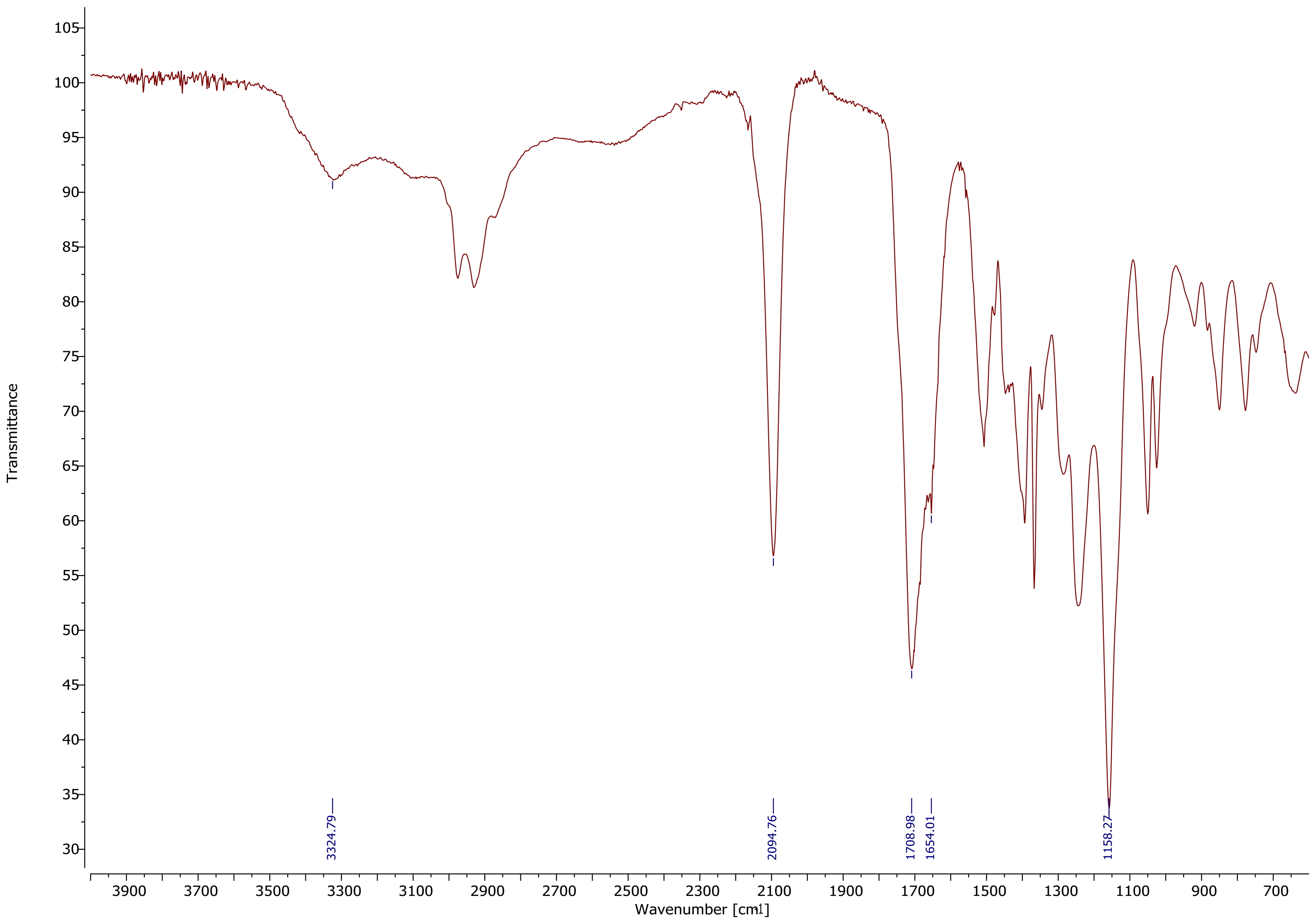

IR spectrum of S-(6-Azidohex-2-yn-1-yl)-N-(tert-butoxycarbonyl)-L-homocysteine (4)

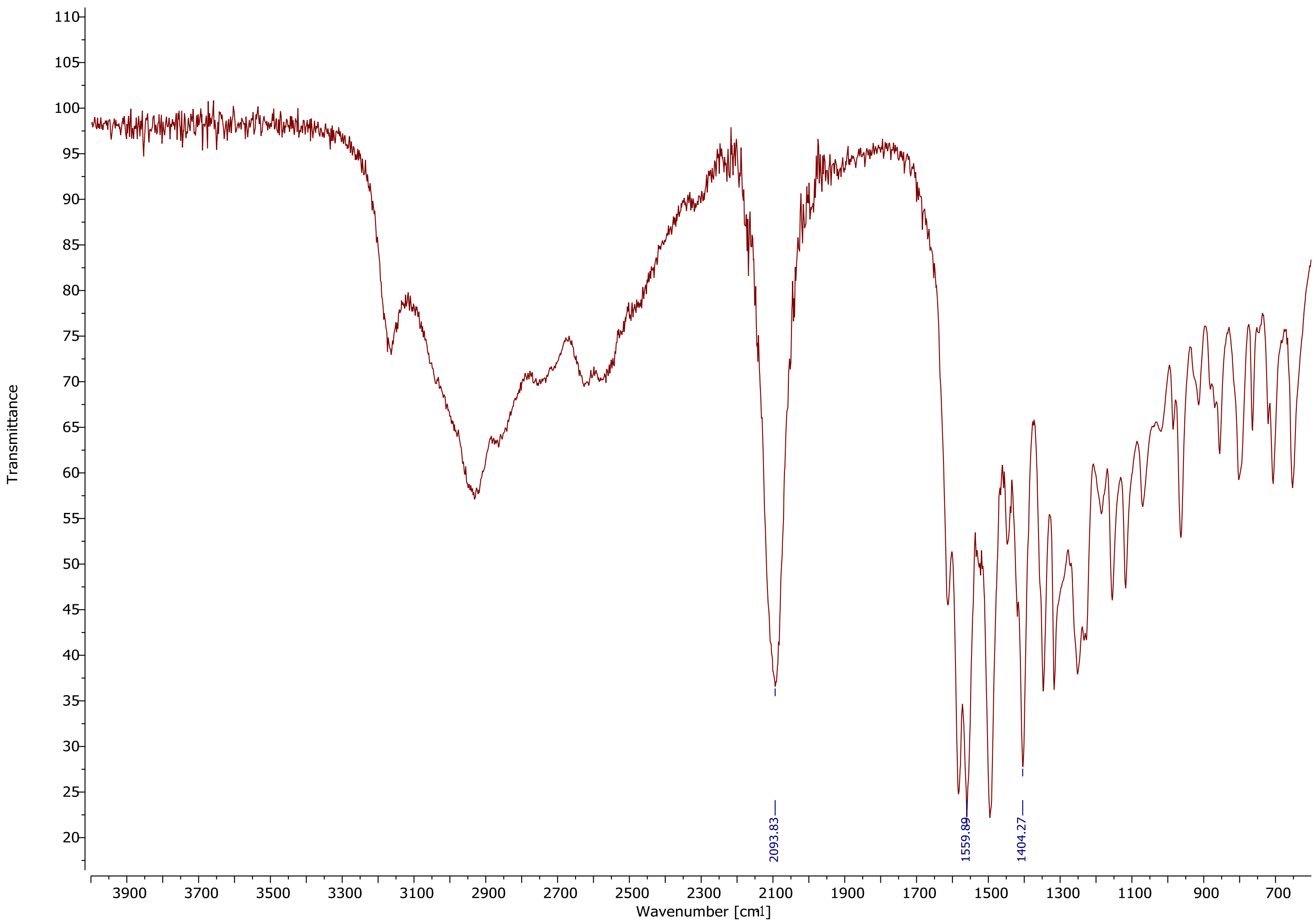

IR spectrum of S-(6-azidohex-2-yn-1-yl)-L-homocysteine zwitterion (5a)

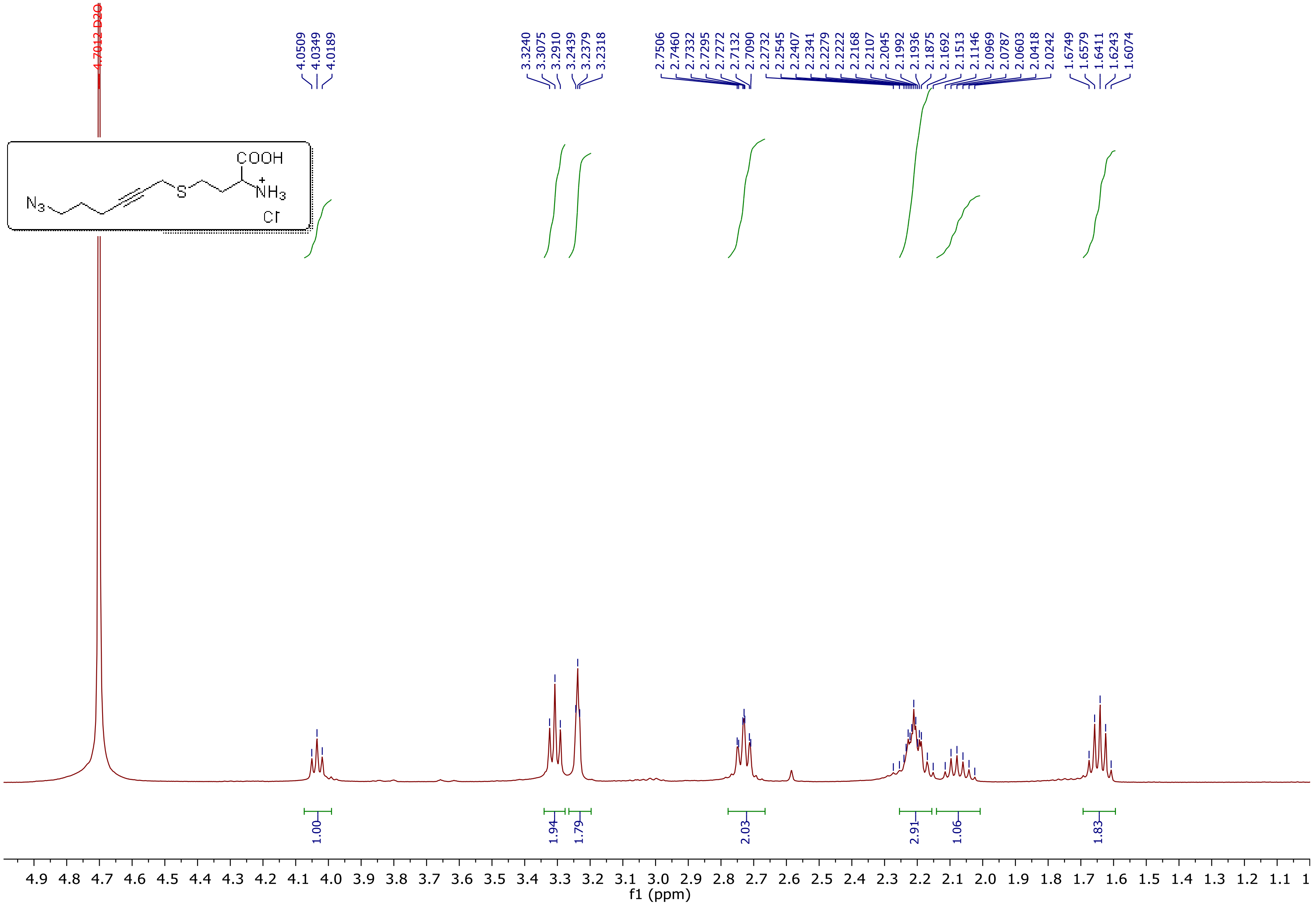

^{1}H NMR spectrum of S-(6-azidohex-2-yn-1-yl)-L-homocysteine chloride (5b)

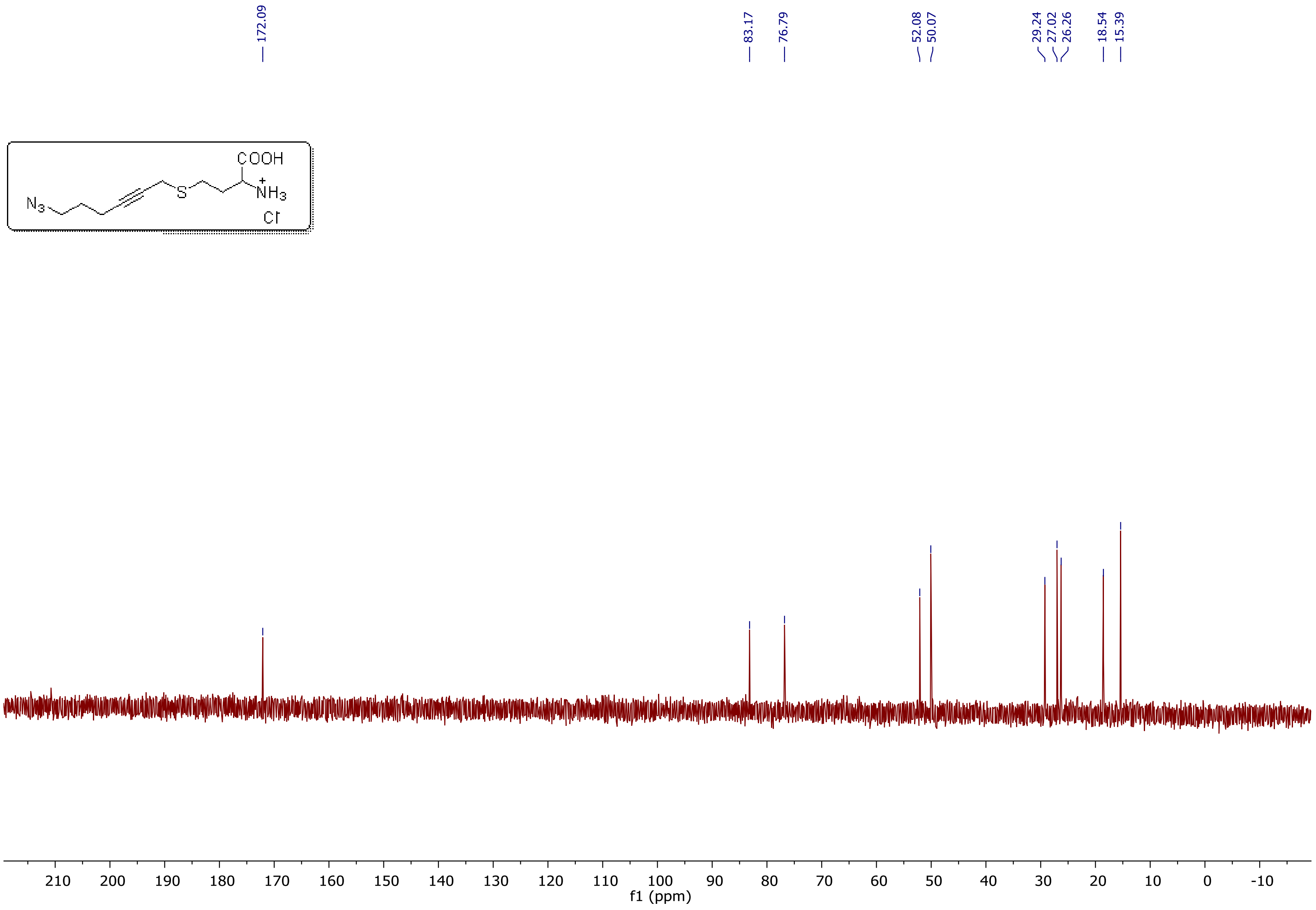

^{13}C NMR spectrum of S-(6-azidohex-2-yn-1-yl)-L-homocysteine chloride (5b)

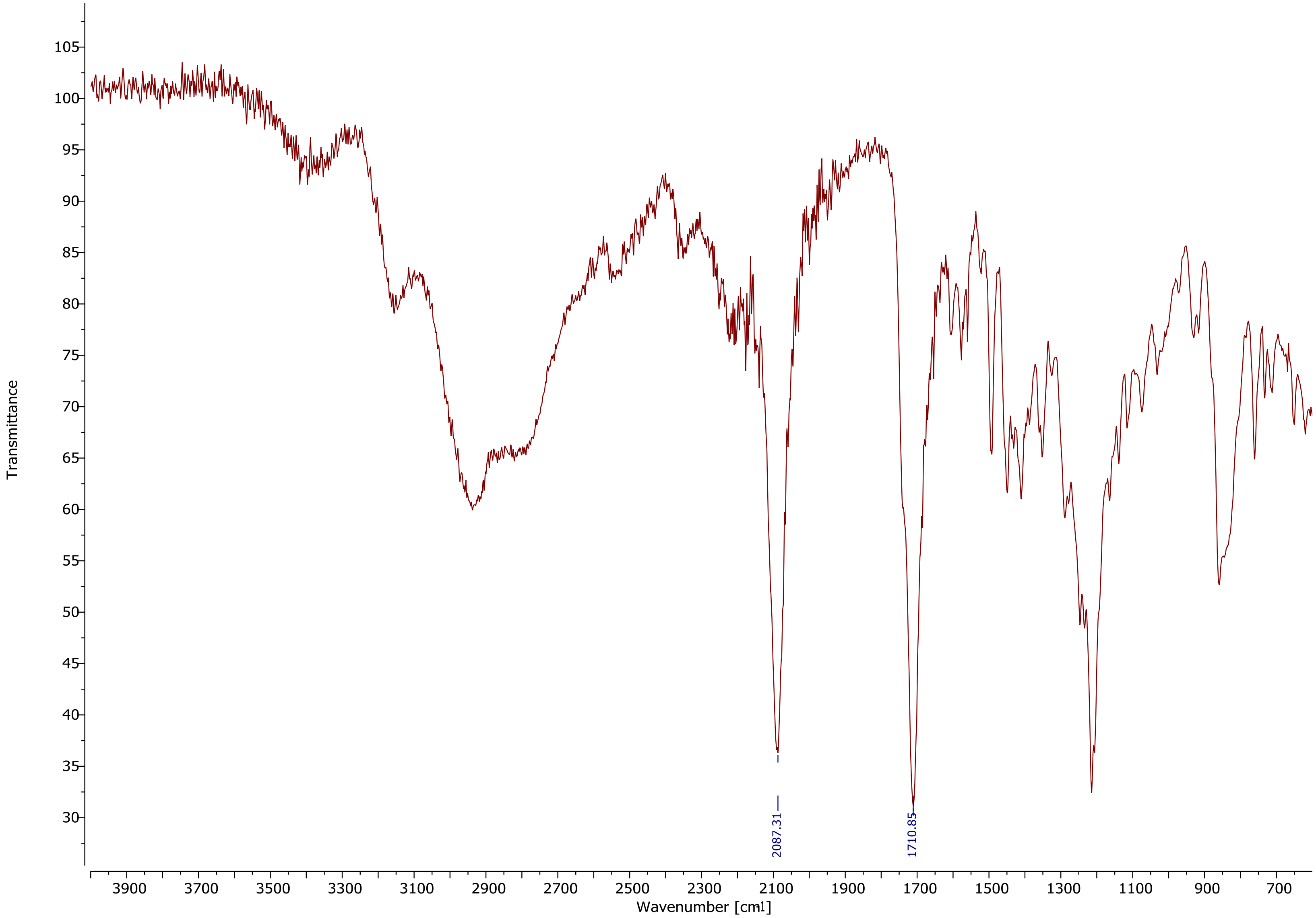

IR spectrum of S-(6-azidohex-2-yn-1-yl)-L-homocysteine chloride (5b)
